# BMI and Mortality in UK Biobank: Revised Estimates Using Mendelian Randomization

**DOI:** 10.1002/oby.22313

**Published:** 2018-10-25

**Authors:** Kaitlin H. Wade, David Carslake, Naveed Sattar, George Davey Smith, Nicholas J. Timpson

**Affiliations:** ^1^ MRC Integrative Epidemiology Unit University of Bristol Bristol UK; ^2^ Population Health Sciences, Bristol Medical School, Faculty of Health Sciences University of Bristol Bristol UK; ^3^ Institute of Cardiovascular and Medical Sciences, British Heart Foundation Glasgow Cardiovascular Research Centre University of Glasgow Glasgow UK

## Abstract

**Objective:**

The aim of this study was to obtain estimates of the causal relationship between BMI and mortality.

**Methods:**

Mendelian randomization (MR) with BMI‐associated genotypic variation was used to test the causal effect of BMI on all‐cause and cause‐specific mortality in UK Biobank participants of White British ancestry.

**Results:**

MR analyses supported a causal association between higher BMI and greater risk of all‐cause mortality (hazard ratio [HR] per 1 kg/m^2^: 1.03; 95% CI: 0.99‐1.07) and mortality from cardiovascular diseases (HR: 1.10; 95% CI: 1.01‐1.19), specifically coronary heart disease (HR: 1.12; 95% CI: 1.00‐1.25) and those excluding coronary heart disease/stroke/aortic aneurysm (HR: 1.24; 95% CI: 1.03‐1.48), stomach cancer (HR: 1.18; 95% CI: 0.87‐1.62), and esophageal cancer (HR: 1.22; 95% CI: 0.98‐1.53), and a decreased risk of lung cancer mortality (HR: 0.96; 95% CI: 0.85‐1.08). Sex stratification supported the causal role of higher BMI increasing bladder cancer mortality risk (males) but decreasing respiratory disease mortality risk (males). The J‐shaped observational association between BMI and mortality was visible with MR analyses, but the BMI at which mortality was minimized was lower and the association was flatter over a larger BMI range.

**Conclusions:**

Results support a causal role of higher BMI in increasing the risk of all‐cause mortality and mortality from several specific causes.

## Introduction

While severe obesity (BMI ≥ 35 kg/m^2^) increases the risk of death, having a BMI > 25 kg/m^2^ also increases the risk of all‐cause mortality and mortality from vascular diseases, diabetes, respiratory diseases, and cancer in a dose‐response manner 1, 2, 3, 4. For example, each 5‐kg/m^2^ higher BMI (or a transition between BMI categories) increased the risk of mortality by > 30%, vascular mortality by 40%, and diabetic, renal, and hepatic mortality by 60% to 120% 1, 5. Additionally, ~3.6% of new adult cancer cases in 2012 (*n* = ~481,000; aged > 30 after 10 years) were attributable to high BMI, a quarter of which could be attributed to rising BMI since 1982 6.

However, there are inconsistencies within the literature relating to the “obesity paradox,” whereby being overweight can appear protective 7, 8. Most prominently, in a systematic review and meta‐analysis (> 2.88 million individuals), Flegal et al. showed ~6% lower risk of all‐cause mortality in individuals with overweight (i.e., BMI 25.0‐29.9 kg/m^2^) compared with normal weight (i.e., BMI 18.5‐24.9 kg/m^2^) 7. Such controversial findings are not without limitation, as confounding by age, ill‐health, and lifestyle as well as bias are likely 9. Furthermore, many studies report a characteristic J‐shaped curve in the association between BMI and mortality 1, 2, 5, 8, 10, in which individuals at the lower tail of the BMI distribution (i.e., underweight [< 18.5 kg/m^2^] or below 22.5‐24.9 kg/m^2^) have an increased risk of mortality along with those above the “normal weight” threshold 1, 2, 5. However, there are discrepancies in the reporting of this pattern, specifically between condition‐specific mortality and in populations of varying ancestries 3, 11, 12, 13.

Mendelian randomization (MR) is a well‐documented application of instrumental variable (IV) methodology using genetic variants (most commonly, single‐nucleotide polymorphisms [SNPs]) as IVs to provide relatively unbiased causal estimates of the effect of an exposure (i.e., BMI) on an outcome (i.e., mortality) 14, 15. MR has provided evidence to support a causal effect of higher BMI increasing the risk of cardiovascular diseases (CVDs), diabetes, cardiometabolic traits, and various cancers 16, 17, 18, 19, 20, 21, 22, 23, 24, 25, 26, 27. However, no study has explicitly used MR to explore the causal role of BMI in all‐cause and cause‐specific mortality. Here, data from the UK Biobank study, a powerful and large resource of comprehensive phenotypic, genetic, and death registry data from the United Kingdom, were used to generate overall and sex‐stratified estimates of the causal role of BMI in all‐cause and cause‐specific mortality. This approach was chosen to reduce problems of confounding and bias (e.g., reporting and recall bias) seen in traditional epidemiological studies.

## Methods

### The UK Biobank study

**Figure 1:**
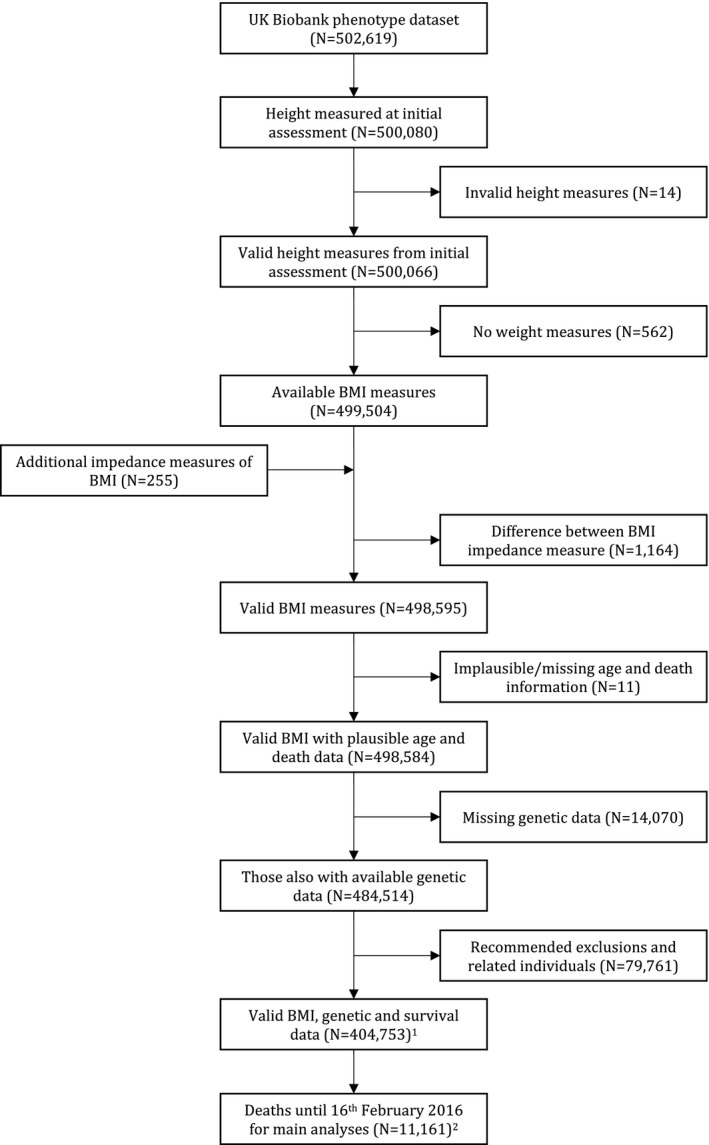
Flowchart of those included in main analyses. Of those with valid BMI, genetic, and survival data, 335,308 were of White British ancestry. Of those who had died by February 16, 2016, 9,570 were of White British ancestry.

UK Biobank recruited more than 500,000 people aged 37 to 73 years (99.5% were 40‐69 years) from the United Kingdom in 2006 to 2010. The study, participants, and quality control have been described previously 28, 29, 30. UK Biobank received ethical approval from the NHS National Research Ethics Service North West (Research Ethics Committee [REC] reference: 11/NW/0382). Details of BMI, mortality, covariables, and genotyping are presented in the online Supporting Information. At the time of this study, and after exclusions based on quality control parameters for phenotypic and genetic data (Supporting Information Figure S1), 335,308 participants of White British ancestry had valid BMI, genetic, and survival data, and 9,750 of these participants had died (Figure 1; Supporting Information Table S1).

### Statistical analysis

Cox proportional hazards regression models were used to estimate hazard ratios (HRs) for all‐cause and cause‐specific mortality per unit increase (kg/m^2) in BMI. The participant’s age was used as a measure of time; thus, models were adjusted for age. Analyses were conducted with the following two models: (1) adjusted for secular trends (date of birth [DOB]) and (2) additionally adjusted for current occupation, qualifications, smoking status, alcohol intake, and physical activity. Analyses were restricted to the conditions responsible for a minimum number of deaths (> 40) 31 and performed in whole and sex‐stratified samples; therefore, results for all‐cause mortality include all individuals who had died by February 16, 2016 (*n *= 9,750), but individual mortality causes presented may not equate to this number (Supporting Information Table S1).

To generate the weighted genetic risk score (GRS) for MR analyses, the dosage of each genetic variant was weighted by its relative effect size on BMI reported by the Genetic Investigation of Anthropometric Traits (GIANT) consortium 32 and summed across all variants (Supporting Information Table S2). The resulting total was rescaled by dividing by the sum of all effect sizes on BMI reported by the GIANT consortium 32 and multiplied by the number of genetic variants used, providing a variable reflecting the number of average BMI‐increasing alleles each participant possessed 33. The associations of the weighted GRS with BMI and of each covariable with BMI and the GRS were tested using linear regression, and associations of each covariable with all‐cause mortality were assessed using Cox proportional hazards regression models. Associations with the GRS were adjusted for the first 10 genetic principal components (PCs).

For MR analyses, the instrumental variable ratio method was conducted. First, BMI was regressed on a GRS comprising 77 SNPs (the denominator of the ratio method estimator), adjusted for the first 10 genetic PCs. Second, Cox proportional hazards models were used to estimate the log (HR) of each mortality outcome per unit increase in the GRS (the numerator of the ratio method estimator), adjusted for secular trends (DOB) and the first 10 genetic PCs. Exponentiation of the resulting ratio of the numerator and denominator yielded an MR estimate of the HR of each mortality outcome per unit increase (kg/m^2) in BMI (Box 1). Confidence intervals (CIs) were obtained using Taylor series expansions 34. A simplification of the matrix method for the Durbin‐Wu‐Hausman (DWH) test for endogeneity was used to compare the HR estimated from conventional Cox regression and MR (online Supporting Information Methods). A priori, conclusions were based on effect estimates and their CIs rather than using an arbitrary *P* value threshold 35. For example, given two effects with the same HR, one with narrow CIs and the other with wider CIs that included the null, both are described as showing the same effect, but one is more imprecisely estimated and should be treated with caution until replicated. All analyses were conducted using Stata software version 15 (StataCorp LLC, College Station, Texas).

Box 1 **Mendelian randomization in the context of survival analyses**

Mendelian randomization (MR) is a well‐documented method that uses genetic variation (most commonly, single‐nucleotide polymorphisms [SNPs] or a genetic risk score [GRS] comprising multiple SNPs) as a proxy for an exposure of interest in an instrumental variable analysis to provide an unbiased and unconfounded causal estimate of the effect of the exposure (here, BMI) on an outcome (here, mortality). MR relies on the following three key assumptions: (1) the instrument (Z) is associated with the exposure (X); (2) the instrument is independent of confounding factors (C) of the association between the exposure (X) and outcome (Y); and (3) there must be no independent pathway between the instrument (Z) and outcome (Y) other than through exposure (X)—horizontal pleiotropy (see Figure).

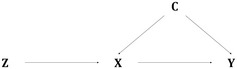

The first MR studies used data from large‐scale cohorts and consortia that had available genetic, exposure, and outcome data in one sample, in which the causal estimate could be calculated in a variety of ways (1). However, having all information available for MR analyses (genetic, exposure, and outcome data) within one sample is difficult in large enough samples for adequate statistical power. More recently, and with the rise in genome‐wide association studies, two‐sample MR methods have been developed to overcome the necessity of having all information within one sample and have proved useful in situations in which both genetic and exposure data are present in one sample and both genetic and outcome data are present in a second sample. Here, the causal estimate can be calculated in the many ways, each of which has different assumptions and provides the ability to test the validity of the MR estimate. For example: inverse variance weighted (2), weighted median‐ and mode‐based estimators (3,4), and MR‐Egger regression (5).

While MR is an established technique within population health sciences, the application in longitudinal studies and survival analyses is new; therefore, there is no “gold standard.” For this manuscript, the instrumental variable ratio estimate was used in primary analyses, separating out the analyses that generated the numerator and denominator:

$$\beta_{\mathrm{IV}}=\beta_{\mathrm{YZ}}/\beta_{\mathrm{XZ}}$$

where $\beta_{\mathrm{IV}}$ is the instrumental variable causal estimate of the association between BMI and mortality; $\beta_{\mathrm{YZ}}$ (numerator) is the log hazard ratio (HR) of each mortality outcome (Y) with each unit increase in a GRS (Z) derived from the Cox proportional hazards model; and $\beta_{\mathrm{XZ}}$ (denominator) is the change in BMI (X) with each unit increase in the GRS (Z). Exponentiating the resulting ratio of the numerator and denominator yielded an MR estimate of the HR of each mortality outcome per unit increase (kg/m^2) in BMI. For primary analyses in the current study, the instrument used was a GRS comprising 77 SNPs associated with BMI reported in the Genetic Investigation of Anthropometric Traits (GIANT) consortium. The GRS was generated in the UK Biobank by weighting the genetic dosage of each of the 77 SNPs by its relative effect size reported in the GIANT consortium, then summed across all SNPs, divided by the combined effect size of all SNPs, and multiplied by the number of SNPs available (*n* = 77). The GRS therefore represented the number of average BMI‐increasing variants that each individual possessed. In sensitivity analyses in this study, each of the 77 SNPs was used individually and combined using the various two‐sample MR techniques (inverse variance weighted, weighted median, weighted mode, and the MR‐Egger estimators) to test the validity of MR assumptions.

For more detail on each method discussed, see the following published articles:

(1)** One‐sample MR methods:** Haycock et al. Best (but oft‐forgotten) practices: the design, analysis and interpretation of Mendelian randomization studies. *Am J Clin Nutr* 2016;103:965‐978.

(2)** Inverse‐variance weighted:** Burgess S, Butterworth A, Thompson SG. Mendelian randomization analysis with multiple genetic variants using summarized data. *Genet Epidemiol* 2013;37:658‐665.

(3)** Weighted median:** Bowden J, Davey Smith G, Haycock PC, Burgess S. Consistent estimation in Mendelian randomization with some invalid instruments using a weighted median estimator. *Genet Epidemiol *2016;40:304‐314.

(4)** Weighted mode:** Hartwig FP, Davey Smith G, Bowden J. Robust inference in summary data Mendelian randomization via the zero modal pleiotropy assumption. *Int J Epidemiol* 2017;46:1985‐1998.

(5)** MR‐Egger:** Bowden J, Davey Smith G, Burgess S. Mendelian randomization with invalid instruments; effect estimation and bias detection through Egger regression. *Int J Epidemiol *2015;44:512‐525.

### Linearity and proportional hazards assumption

Cubic spline models for both BMI (adjusted for variables in model 2 described above) and the GRS (adjusted for secular trends [DOB] and the first 10 genetic PCs) were plotted to test their pattern of association with mortality. Linearity tests were conducted after removing data below or above the 1st or 99th percentile, respectively, because of the scarcity of data toward the tails of the BMI distribution. In addition, an approximate MR analogue to the nonlinear plot of mortality against BMI was obtained by estimating localized average causal effects (i.e., MR estimates of the log‐linear effect of BMI on mortality, adjusted for secular trends [DOB] and the first 10 genetic PCs) within percentiles (the 5th, 10th, 25th, 50th, 75th, and 85th percentile) of the instrument‐free exposure (i.e., BMI that is orthogonal to the GRS) 36. These localized average causal effects were joined and plotted against corresponding quantiles of the original exposure 37. HRs were calculated relative to the mean BMI (27 kg/m^2^), and CIs were obtained using bootstrapping (*n* = 1,000). Meta‐regression was used to test for a linear trend in the GRS‐BMI association (i.e., denominator of the ratio method) over quantiles of the instrument‐free exposure.

To check the proportional hazards assumption, Schoenfeld residuals for BMI from the cubic spline models of each mortality outcome were tested for association with rank‐normalized natural log of the follow‐up time (age) using both Cox regression and MR (132 tests in the whole sample and sex‐stratified analyses for both methodologies) using Pearson correlations. If there was evidence for an association (using a Bonferroni‐corrected α level of 0.05/132 = 0.0004), an interaction term was fitted to the cubic spline model using the “tvc()” option in Stata.

### Sensitivity analysis

Sensitivity analyses were conducted to (i) investigate the validity of the GRS as an IV using the MR‐Egger 38, weighted median, and mode‐based estimators 39 compared with the inverse‐variance weighted method for two‐sample MR 38, 40; (ii) evaluate the impact of covariables associated with the GRS; and (iii) explore the sensitivity of the GRS by excluding genetic variants implicated as pleiotropic (*n* = 7; leaving 70 SNPs; Supporting Information Table S3) 17, 33. Details are presented in the online Supporting Information.

## Results

Included participants had an average age (at initial assessment) of 56.9 years (SD 8.0) and BMI of 27.4 (SD 4.7) (Table 1). Of the 335,308 participants with required information for mortality analyses, 9,570 participants (*n* = 5,882/3,688 males/females, respectively) had died by February 16, 2016, at an average age of 65.7 years old (SD 6.9) from various CVDs and cancers (Supporting Information Table S1‐S1b).

**Table 1:** Descriptive statistics for UK Biobank participants of White British ancestry included in the main analyses

Variable	*n*	Mean (SD) or percentage
**Age (y) at initial assessment**	335,308	56.87 (8.00)
**Sex (% of males)**	335,308	46.22
**BMI (kg/m^2^)**	335,308	27.38 (4.74)
**Smoking status**	334,142	54.82
Never	183,170	35.27
Former	117,838	9.92
Current	33,134	
**Alcohol drinker status**	335,074	
Never	10,311	3.08
Former	11,368	3.39
Current	313,395	93.53
**Highest qualifications**	275,544	
College or university degree	106,280	38.57
A‐levels	38,271	13.89
O‐levels	73,770	26.77
CSEs	18,016	6.54
NVQ/HND/HNC	22,012	7.99
Other professional qualifications	17,195	6.24
**Current employment status**	332,835	
In paid employment or self‐employed	190,085	57.11
Retired	117,615	35.34
Looking after home/family	8,690	2.61
Unable to work because of sickness/disability	9,982	3.00
Unemployed	4,436	1.33
Doing unpaid or voluntary work	1,404	0.42
Full‐ or part‐time student	623	0.19
**Days/week spent doing vigorous physical activity**	319,813	1.82 (1.94)
**Genotyping chip** a	335,308	9.24
**Age at death (y)**	9,570	65.66 (6.88)
**Date of death** b	9,570	06/02/2013 (07/07/2007‐02/16/2016)

a UK BiLEVE participants genotyped on Affymetrix Axiom Array.

b Recorded as mean (minimum and maximum) date of death.

CSE, certificate of secondary education; HNC, higher national certificate; HND, higher national diploma; NVQ, national vocational qualification.

### Observational analyses

Cox regression models provided evidence that BMI was associated with a higher risk of all‐cause mortality (HR per 1‐kg/m^2^ higher BMI: 1.02; 95% CI: 1.02‐1.03) and mortality from CVD (HR: 1.07; 95% CI: 1.06‐1.08), specifically CHD (HR: 1.08; 95% CI: 1.06‐1.09) and those excluding CHD/stroke/aortic aneurysm (HR: 1.10; 95% CI: 1.08‐1.12), alongside mortality from overall cancer (HR: 1.01; 95% CI: 1.00‐1.02) and cancers of the stomach (HR: 1.05; 95% CI: 1.01‐1.09), esophagus (HR: 1.03; 95% CI: 1.00‐1.06), kidney (HR: 1.07; 95% CI: 1.04‐1.11), and liver (HR: 1.05; 95% CI: 1.02‐1.09) (Table 2). There was evidence of an inverse association between BMI and lung cancer mortality (HR: 0.97; 95% CI: 0.95‐0.99). There was also weak evidence to suggest that higher BMI marginally increased mortality from stroke, aortic aneurysm, and cancers of the colorectum, pancreas, and brain while decreasing mortality from respiratory diseases, bladder cancer, malignant melanoma, and external causes (but estimates had wide CIs).

**Table 2:** Observational and MR analyses of all‐cause and cause‐specific mortality by BMI in UK Biobank participants of White British ancestry (men and women)

Cause of death	*n* a	Observational						DWH^b^
		Unadjusted		Adjusted		MR analyses		
		HR (95% CI)^b^	*P*	HR (95% CI)[Link]	*P*	HR (95% CI)^a^	*P*	
**All‐cause** c	9,570	1.03 (1.02‐1.03)	1.16 × 10^‐35^	1.02 (1.02‐1.03)	1.20 × 10^‐14^	1.03 (0.99‐1.07)	0.17	0.96
**Cardiovascular disease** c	1,967	1.07 (1.06‐1.08)	1.67 × 10^‐65^	1.07 (1.06‐1.08)	3.15 × 10^‐38^	1.10 (1.01‐1.19)	0.04	0.62
**Coronary heart disease**	1,087	1.07 (1.06‐1.09)	3.16 × 10^‐40^	1.08 (1.06‐1.09)	1.35 × 10^‐25^	1.12 (1.00‐1.25)	0.06	0.51
**Stroke**	346	1.02 (1.00‐1.04)	0.12	1.01 (0.98‐1.04)	0.53	0.98 (0.80‐1.20)	0.84	0.70
**Aortic aneurysm**	109	1.03 (0.99‐1.07)	0.10	1.03 (0.98‐1.08)	0.32	0.80 (0.56‐1.15)	0.23	0.17
**Other cardiovascular diseases**	425	1.11 (1.09‐1.13)	1.19 × 10^‐40^	1.10 (1.08‐1.12)	5.74 × 10^‐22^	1.24 (1.03‐1.48)	0.02	0.23
**Respiratory diseases**	532	1.00 (0.98‐1.01)	0.65	0.98 (0.96‐1.01)	0.19	1.03 (0.88‐1.22)	0.68	0.64
**Cancer** c	5,613	1.01 (1.01‐1.02)	1.53 × 10^‐06^	1.01 (1.00‐1.02)	0.01	0.99 (0.94‐1.04)	0.68	0.34
**Lung cancer**	993	0.99 (0.97‐1.00)	0.10	0.97 (0.95‐0.99)	0.01	0.96 (0.85‐1.08)	0.49	0.62
**Colorectal cancer**	552	1.01 (1.00‐1.03)	0.14	1.01 (0.99‐1.04)	0.18	1.06 (0.90‐1.25)	0.46	0.56
**Pancreatic cancer**	388	1.01 (0.99‐1.03)	0.45	1.01 (0.99‐1.04)	0.34	1.10 (0.91‐1.33)	0.34	0.38
**Stomach cancer**	144	1.06 (1.03‐1.09)	0.0003	1.05 (1.01‐1.09)	0.03	1.18 (0.87‐1.62)	0.29	0.48
**Esophageal cancer**	283	1.04 (1.01‐1.06)	0.002	1.03 (1.00‐1.06)	0.05	1.22 (0.98‐1.53)	0.08	0.15
**Malignant melanoma**	119	1.00 (0.97‐1.04)	0.86	0.98 (0.93‐1.03)	0.38	1.18 (0.83‐1.66)	0.36	0.36
**Kidney cancer**	181	1.08 (1.05‐1.11)	1.94 × 10^‐09^	1.07 (1.04‐1.11)	3.41 × 10^‐05^	0.93 (0.71‐1.23)	0.62	0.30
**Bladder cancer**	101	1.02 (0.98‐1.06)	0.40	0.97 (0.92‐1.03)	0.33	0.79 (0.54‐1.15)	0.21	0.18
**Brain cancer**	280	1.01 (0.99‐1.04)	0.37	1.01 (0.98‐1.04)	0.46	1.02 (0.81‐1.27)	0.89	0.97
**Liver cancer**	169	1.07 (1.04‐1.10)	1.04 × 10^‐06^	1.05 (1.02‐1.09)	0.005	0.99 (0.74‐1.32)	0.95	0.60
**Lymphatic cancer**	528	1.00 (0.98‐1.02)	0.88	1.00 (0.98‐1.02)	0.87	1.04 (0.88‐1.22)	0.67	0.68
**Other cancers**	755	1.00 (0.99‐1.02)	0.92	1.00 (0.98‐1.02)	0.87	0.95 (0.83‐1.09)	0.46	0.45
**External causes**	306	0.99 (0.97‐1.01)	0.44	0.97 (0.95‐1.00)	0.07	1.30 (1.05‐1.61)	0.02	0.01

a Number of deaths from all causes or cause‐specific mortality.

b Adjusted for secular trends (date of birth); estimates represent HR with each unit increase in BMI (kg/m^2^).

Adjusted for secular trends (date of birth), highest household occupation, education, smoking status, alcohol intake, and physical activity.

Adjusted for secular trends (date of birth) and first 10 genetic principal components.

*P* value for comparing estimates derived from observational and MR analyses using simplification of matrix method for DWH test statistic (see Supporting Information Methods).

c Total number of UK Biobank participants who had died by February 16, 2016, from any cause (or those specifically defined as cardiovascular disease or cancer), which was stratified further into primary diseases of focus (excluding mortality causes with fewer than 40 deaths and all other causes; see Supporting Information Table S1a).

DWH, Durbin‐Wu‐Hausman; MR, Mendelian randomization.

In males, results were similar to the whole sample but with additional evidence for an association between higher BMI and decreased respiratory disease mortality (HR: 0.91; 95% CI: 0.88‐0.95), which was weaker in the overall sample, an increased prostate cancer mortality (HR: 1.05; 95% CI: 1.02‐1.08), greater magnitudes of association of higher BMI with a decreased risk of mortality from lung cancer (HR: 0.94; 95% CI: 0.91‐0.97) and bladder cancer (HR: 0.93; 95% CI: 0.86‐1.00), and increased risk of mortality from esophageal cancer (HR: 1.07; 95% CI: 1.03‐1.11) and liver cancer (HR: 1.08; 95% CI: 1.03‐1.13) (Table 3). The estimate of association between BMI and brain cancer mortality was in the reverse direction to that obtained in the whole sample but with wide CIs.

**Table 3:** Observational and MR analyses of all‐cause and cause‐specific mortality by BMI in male UK Biobank participants of White British ancestry

Cause of death	*n* ^a^	Observational				MR analyses		DWH^c^
		Unadjusted		Adjusted				
		HR (95% CI)^b^	*P*	HR (95% CI)^a^	*P*	HR (95% CI)^b^	*P*	
**All‐cause** d	5,882	1.03 (1.02‐1.03)	4.00 × 10^‐18^	1.02 (1.01‐1.03)	1.59 × 10^‐07^	1.03 (0.98‐1.08)	0.26	0.93
**Cardiovascular disease** d	1,467	1.08 (1.07‐1.09)	1.23 × 10^‐48^	1.08 (1.06‐1.09)	7.39 × 10^‐28^	1.09 (0.98‐1.20)	0.10	0.88
**Coronary heart disease**	906	1.08 (1.07‐1.09)	8.84 × 10^‐32^	1.08 (1.06‐1.10)	4.80 × 10^‐18^	1.12 (0.98‐1.27)	0.09	0.62
**Stroke**	194	1.03 (0.99‐1.06)	0.10	1.02 (0.98‐1.07)	0.29	1.01 (0.76‐1.33)	0.96	0.88
**Aortic aneurysm**	83	1.04 (0.99‐1.09)	0.14	1.03 (0.97‐1.09)	0.40	0.80 (0.52‐1.21)	0.29	0.22
**Other cardiovascular diseases**	284	1.11 (1.09‐1.14)	9.96 × 10^‐25^	1.11 (1.08‐1.14)	9.99 × 10^‐15^	1.16 (0.92‐1.45)	0.21	0.75
**Respiratory diseases**	361	0.94 (0.91‐0.97)	1.08 × 10^‐05^	0.91 (0.88‐0.95)	2.22 × 10^‐06^	1.04 (0.85‐1.27)	0.71	0.32
**Cancer** d	3,113	1.01 (1.00‐1.02)	0.002	1.01 (1.00‐1.02)	0.06	1.00 (0.93‐1.07)	0.98	0.72
**Lung cancer**	571	0.96 (0.94‐0.98)	0.0002	0.94 (0.91‐0.97)	4.27 × 10^‐05^	0.92 (0.78‐1.08)	0.29	0.57
**Prostate cancer**	308	1.03 (1.01‐1.06)	0.01	1.05 (1.02‐1.08)	0.004	0.87 (0.70‐1.08)	0.21	0.12
**Colorectal cancer**	329	1.03 (1.00‐1.05)	0.04	1.02 (0.99‐1.05)	0.23	1.09 (0.88‐1.34)	0.43	0.59
**Pancreatic cancer**	201	1.01 (0.97‐1.04)	0.76	1.00 (0.96‐1.04)	0.97	1.18 (0.90‐1.54)	0.24	0.25
**Stomach cancer**	105	1.07 (1.03‐1.11)	0.001	1.06 (1.01‐1.12)	0.02	1.15 (0.79‐1.68)	0.45	0.70
**Esophageal cancer**	226	1.06 (1.03‐1.09)	1.06 × 10^‐04^	1.07 (1.03‐1.11)	1.17 × 10^‐04^	1.28 (0.99‐1.65)	0.06	0.14
**Malignant melanoma**	78	0.99 (0.94‐1.05)	0.85	0.97 (0.91‐1.04)	0.42	0.99 (0.64‐1.53)	0.96	0.98
**Kidney cancer**	137	1.09 (1.05‐1.12)	8.27 × 10^‐07^	1.08 (1.03‐1.13)	5.72 × 10^‐04^	1.04 (0.75‐1.44)	0.82	0.79
**Bladder cancer**	78	0.98 (0.93 ‐1.04)	0.58	0.93 (0.86 ‐1.00)	0.05	0.73 (0.47 ‐1.13)	0.16	0.18
**Brain cancer**	169	1.01 (0.97‐1.05)	0.59	0.98 (0.94‐1.03)	0.47	1.15 (0.85‐1.54)	0.36	0.39
**Liver cancer**	100	1.11 (1.07‐1.15)	3.18 × 10^‐08^	1.08 (1.03‐1.13)	0.003	1.03 (0.70‐1.52)	0.86	0.73
**Lymphatic cancer**	329	1.00 (0.97‐1.03)	0.91	1.01 (0.98‐1.04)	0.58	1.03 (0.83‐1.27)	0.81	0.79
**Other cancers**	460	0.99 (0.96‐1.01)	0.22	1.00 (0.98‐1.03)	0.88	0.89 (0.74‐1.06)	0.20	0.26
**External causes**	206	0.97 (0.94‐1.01)	0.12	0.97 (0.93‐1.01)	0.11	1.11 (0.85‐1.45)	0.44	0.32

Number of deaths from all causes or cause‐specific mortality.

Adjusted for secular trends (date of birth); estimates represent HR with each unit increase in BMI (kg/m^2^).

a Adjusted for secular trends (date of birth), highest household occupation, education, smoking status, alcohol intake, and physical activity.

b Adjusted for secular trends (date of birth) and first 10 genetic principal components.

c *P* value for comparing estimates derived from observational and MR analyses using simplification of matrix method for DWH test statistic (see Supporting Information Methods).

d Total number of male UK Biobank participants who had died by February 16, 2016, from any cause (or those specifically defined as cardiovascular disease or cancer), which was stratified further into primary diseases of focus (excluding mortality causes with fewer than 40 deaths and all other causes; see Supporting Information Table S1b).

DWH, Durbin‐Wu‐Hausman; MR, Mendelian randomization.

In females, results were similar to those in the whole sample but with additional evidence for an association between higher BMI and an increased respiratory disease mortality (HR: 1.06; 95% CI: 1.02‐1.10), the estimate of which was in the opposite direction in both the whole sample and males (Table 4). There was also evidence for an association between higher BMI and an increased risk of mortality from endometrial cancer (HR: 1.12; 95% CI: 1.07‐1.18) and both overall and postmenopausal breast cancer (HR: 1.02; 95% CI: 1.00‐1.04). There was no strong evidence of an association of BMI with lung cancer mortality, and the estimate of association between higher BMI and esophageal cancer mortality was in the opposite direction to that observed in the whole sample (HR: 0.87; 95% CI: 0.80‐0.95); however, all CIs overlapped.

**Table 4:** Observational and MR analyses of all‐cause and cause‐specific mortality by BMI in female UK Biobank participants of White British ancestry

Cause of death	*n* a	Observational				MR analyses		DWH^e^
		Unadjusted		Adjusted				
		HR (95% CI)^b^	*P*	HR (95% CI)^c^	*P*	HR (95% CI)^d^	*P*	
**All‐cause** f	3,688	1.02 (1.01‐1.03)	1.84×10^‐11^	1.02 (1.01‐1.02)	3.10 × 10^‐05^	1.03 (0.96‐1.09)	0.42	0.90
**Cardiovascular disease** f	500	1.06 (1.04‐1.08)	6.64 × 10^‐14^	1.06 (1.04‐1.08)	1.55 × 10^‐08^	1.12 (0.95‐1.32)	0.19	0.53
**Coronary heart disease**	181	1.06 (1.03‐1.09)	5.05 × 10^‐06^	1.08 (1.04‐1.12)	6.87 × 10^‐06^	1.12 (0.85‐1.47)	0.43	0.71
**Stroke**	152	1.01 (0.97‐1.04)	0.75	1.00 (0.95‐1.04)	0.84	0.95 (0.70‐1.28)	0.72	0.70
**Other cardiovascular diseases**	141	1.11 (1.08‐1.14)	2.01 × 19^17^	1.10 (1.06‐1.13)	8.41 × 10^‐09^	1.42 (1.04‐1.93)	0.03	0.12
**Respiratory diseases**	171	1.05 (1.02‐1.08)	0.0004	1.06 (1.02‐1.10)	0.002	1.02 (0.77‐1.36)	0.88	0.86
**Cancer** f	2,500	1.01 (1.00‐1.02)	0.01	1.01 (1.00‐1.02)	0.20	0.98 (0.91‐1.05)	0.52	0.35
**Lung cancer**	422	1.01 (0.99‐1.03)	0.53	1.00 (0.97‐1.03)	0.97	1.02 (0.85‐1.22)	0.86	0.91
**Breast cancer**	468	1.02 (1.00‐1.03)	0.05	1.02 (1.00‐1.04)	0.13	0.83 (0.70‐0.99)	0.03	0.02
**Premenopausal**	48	1.00 (0.95‐1.06)	0.94	1.00 (0.95‐1.06)	0.90	0.77 (0.45‐1.32)	0.35	0.34
**Postmenopausal**	420	1.02 (1.00‐1.04)	0.04	1.02 (1.00‐1.04)	0.13	0.84 (0.70‐1.00)	0.05	0.03
**Colorectal cancer**	223	0.99 (0.97‐1.02)	0.59	1.01 (0.98‐1.04)	0.73	1.02 (0.80‐1.31)	0.85	0.80
**Pancreatic cancer**	187	1.01 (0.98‐1.04)	0.57	1.02 (0.99‐1.05)	0.27	1.02 (0.78‐1.34)	0.88	0.93
**Ovarian cancer**	211	1.00 (0.97‐1.02)	0.76	1.00 (0.96‐1.03)	0.82	1.19 (0.92‐1.53)	0.19	0.17
**Endometrial cancer**	50	1.10 (1.06‐1.15)	3.29 × 10^‐06^	1.12 (1.07‐1.18)	1.23 × 10^‐05^	0.63 (0.38‐1.07)	0.09	0.04
**Esophageal cancer**	57	0.95 (0.90‐1.01)	0.10	0.87 (0.80‐0.95)	0.001	1.04 (0.64‐1.70)	0.87	0.71
**Malignant melanoma**	41	1.00 (0.94‐1.06)	0.95	0.97 (0.90‐1.04)	0.40	1.61 (0.91‐2.87)	0.10	0.10
**Kidney cancer**	44	1.08 (1.03‐1.13)	0.002	1.06 (1.00‐1.13)	0.06	0.67 (0.38‐1.17)	0.16	0.09
**Brain cancer**	111	1.00 (0.97‐1.04)	0.80	1.03 (0.99‐1.07)	0.19	0.85 (0.60‐1.21)	0.36	0.34
**Liver cancer**	69	1.03 (0.99‐1.08)	0.18	1.03 (0.97‐1.09)	0.34	0.94 (0.60‐1.46)	0.77	0.67
**Lymphatic cancer**	199	1.00 (0.97‐1.02)	0.76	0.98 (0.94‐1.01)	0.21	1.05 (0.81‐1.37)	0.70	0.68
**Other cancers**	295	1.01 (0.98‐1.03)	0.52	0.99 (0.96‐1.02)	0.60	1.04 (0.84‐1.29)	0.71	0.76
**External causes**	100	0.99 (0.95‐1.03)	0.72	0.96 (0.91‐1.01)	0.09	1.79 (1.23‐2.58)	0.002	0.002

a Number of deaths from all causes or cause‐specific mortality.

b Adjusted for secular trends (date of birth); estimates represent HR with each unit increase in BMI (kg/m^2^).

c Adjusted for secular trends (date of birth), highest household occupation, education, smoking status, alcohol intake, and physical activity.

d Adjusted for secular trends (date of birth) and first 10 genetic principal components.

e *P* value for comparing estimates derived from observational and MR analyses using simplification of matrix method for DWH test statistic (see Supporting Information Methods).

f Total number of female UK Biobank participants who had died by February 16, 2016, from any cause (or those specifically defined as cardiovascular disease or cancer), which was stratified further into primary diseases of focus (excluding mortality causes with fewer than 40 deaths and all other causes; see Supporting Information Table S1b).

DWH, Durbin‐Wu‐Hausman; MR, Mendelian randomization.

### Association between GRS and BMI

Each unit increase in the GRS (comprising 77 SNPs) in the UK Biobank participants of White British ancestry was associated with 0.111‐kg/m^2^ higher BMI (95% CI: 0.109‐0.114), explaining 1.8% of the variance, and was slightly greater in females compared with males (Table 5).

**Table 5:** Association between weighted GRS (comprising 77 SNPs) and BMI in UK Biobank participants of White British ancestry

**Sample**	*n*	Effect estimate (95% CI)^a^	*P*	*R* ^2^ (%)^b^
**Whole sample**	335,308	0.111 (0.109‐0.114)	<1.20 × 10^‐307^	1.82
**Males**	154,967	0.105 (0.101‐0.109)	<1.20 × 10^‐307^	2.06
**Females**	180,341	0.117 (0.112‐0.121)	<1.20 × 10^‐307^	1.70

a Effect estimate (and corresponding *P* value) represents change in BMI (kg/m^2^) per BMI‐increasing allele in individuals of White British ancestry adjusted for first 10 genetic principal components.

b Variance in BMI explained by GRS.

GRS, genetic risk score; SNP, single‐nucleotide polymorphism.

### Covariable analysis

Both BMI and mortality were associated with all covariables, including initial assessment age, sex, smoking status, alcohol consumption, qualifications, employment status, and physical activity (Supporting Information Table S4 and Table S5 for BMI and all‐cause mortality, respectively). Unlike the direct measurement of BMI, the GRS was associated with covariables to a much lesser extent, with all estimates near zero (Supporting Information Table S6).

### MR analyses

Within the whole UK Biobank sample, MR analyses provided estimates of a similar or greater magnitude to observational analyses (with wider CIs), supporting the causal role of higher BMI in increasing the risk of all‐cause mortality (HR: 1.03; 95% CI: 0.99‐1.07) and mortality from CVD (HR: 1.10; 95% CI: 1.01‐1.19), specifically CHD (HR: 1.12; 95% CI: 1.00‐1.25) and those excluding CHD/stroke/aortic aneurysm (HR: 1.24; 95% CI: 1.03‐1.48), alongside mortality from stomach cancer (HR: 1.18; 95% CI: 0.87‐1.62) and esophageal cancer (HR: 1.22; 95% CI: 0.98‐1.53) (Table 2). Although CIs were wide, the effect estimate for higher BMI on decreasing lung cancer mortality was consistent with that obtained in observational analyses (HR: 0.96; 95% CI: 0.85‐1.08). There was also evidence supporting the causal role of higher BMI in increasing mortality from external causes (HR: 1.30; 95% CI: 1.05‐1.61), unlike the inverse association obtained in observational analyses (DWH *P* = 0.01 for comparison). In contrast, the effect estimates for higher BMI on mortality from cancer, kidney cancer, and liver cancer were attenuated or in the opposite direction, with CIs too wide for conclusive interpretation (Table 2).

Results for males were similar to those in the whole sample, as estimates of the causal role of higher BMI in increasing the risk of all‐cause mortality and mortality from all CVDs, stomach cancer, esophageal cancer, and kidney cancer, as well as the decreased risk of mortality from lung cancer and bladder cancer, were consistent to or greater than the observational analyses (Table 3). The effect estimates for higher BMI on mortality from respiratory diseases, cancer, prostate cancer, and liver cancer were attenuated or in the opposite direction, with CIs too wide for conclusive interpretation (Table 3).

In females, the effect estimates of higher BMI increasing the risk of all‐cause mortality and mortality from all CVDs were consistent to the observational analyses (Table 4). The effect estimates for higher BMI on the risk of mortality from breast cancer (HR: 0.83; 95% CI: 0.70‐0.99), specifically postmenopausal breast cancer (HR: 0.84; 95% CI: 0.70‐1.00), endometrial cancer (HR: 0.63; 95% CI: 0.38‐1.07), and external causes (HR: 1.79; 95% CI: 1.23‐2.58) were in the opposite direction of those obtained in observational analyses (DWH *P* = 0.02, 0.03, 0.04, and 0.002, respectively). Furthermore, the effect estimates for higher BMI on mortality from respiratory diseases, overall cancer, esophageal cancer, and kidney cancer were attenuated or in the opposite direction compared with observational analyses but with CIs too wide for conclusive interpretation (Table 4).

While there was some evidence for an observational relationship between higher BMI and mortality from other causes, CIs were too wide for conclusive interpretation in both adjusted observational and MR analyses as well as with sex stratification (Supporting Information Table S7).

### Linearity and proportional hazards assumption

**Figure 2:**
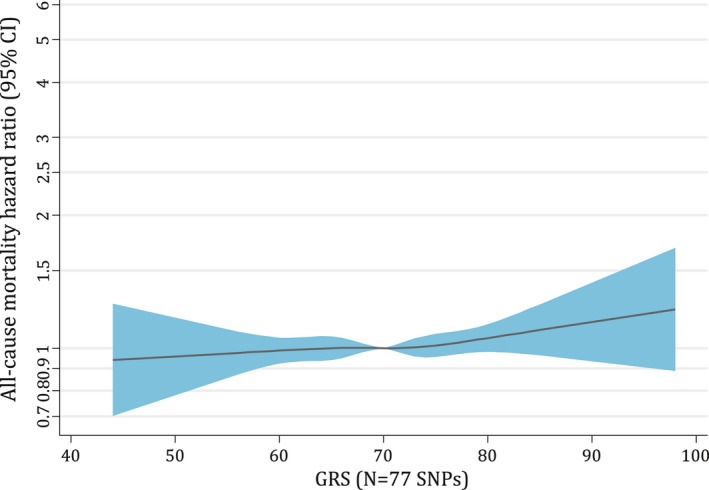
Assessment of linearity in associations of the GRS (comprising 77 SNPs) and all‐cause mortality in the UK Biobank sample of White British ancestry. Association between the GRS (comprising 77 SNPs) and all‐cause mortality, adjusted for secular trends (date of birth) and the first 10 genetic principal components. Linearity tests were conducted after removing data below or above the 1st or 99th percentile of BMI because of the scarcity of data toward the tails of the BMI distribution. Hazard ratios (HRs) were calculated relative to the mean GRS value with 1,000 bootstrap resamples to obtain 95% confidence intervals (CIs). The black lines represent the fitted HRs from cubic spline models (with the mean value of the GRS as the reference). GRS, genetic risk score; SNPs, single‐nucleotide polymorphisms.

**Figure 3:**
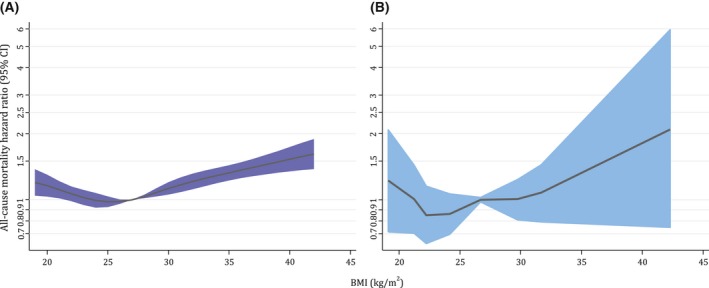
(**A**) Assessment of linearity in associations of BMI and all‐cause mortality in the UK Biobank sample of White British ancestry using BMI. Observational associations between BMI and all‐cause mortality obtained using conventional Cox regression adjusted for secular trends (date of birth), current occupation, qualifications, smoking status, alcohol intake, and physical activity. (**B**) Assessment of linearity in associations of BMI and all‐cause mortality in the UK Biobank sample of White British ancestry using instrument‐free BMI. Approximate analogue using MR stratified by categories of the instrument‐free exposure (divided at the 5th, 10th, 25th, 50th, 75th, and 85th percentile) adjusted for secular trends (date of birth) and first 10 genetic principal components. Localized average causal effects were then joined together and plotted against the corresponding percentiles of the original exposure. Linearity tests were conducted after removing data below or above the 1st or 99th percentile, respectively, because of the scarcity of data toward the tails of the BMI distribution. Hazard ratios (HRs) were calculated relative to the mean BMI (27 kg/m^2^), with 1,000 bootstrap resamples to obtain 95% confidence intervals (CIs). The black lines represent the fitted HRs from cubic spline models (with mean BMI as the reference).

The pattern of the GRS‐mortality association appeared linear (Figure 2); however, the CIs were wide. The observational BMI‐mortality relationship showed evidence of a J‐shaped association (Figure 3A). The J‐shaped BMI‐mortality association remained in MR analyses (Figure 3B) but with a smaller value of BMI at which mortality risk was lowest (~23 vs. ~26 kg/m^2^ with observational analyses) and apparently flatter over a larger BMI range. Meta‐regression provided some evidence that the GRS‐BMI association was nonlinear (*P* = 0.08 for linear trend and *P* < 0.001 for heterogeneity). This was primarily driven by the extreme quantiles of BMI, as removal of these quantiles indicated a linear association (*P* = 0.999 for linear trend and *P* < 0.001 for heterogeneity).

The proportional hazards assumption held for all mortality causes in both the conventional Cox regression and the MR analyses (Supporting Information Table S8a‐S8b for observational and MR analyses, respectively).

### Sensitivity analyses

Across all methods, which assume linearity (including the inverse‐variance weighted method, MR‐Egger, weighted median‐ and mode‐based estimators), MR‐derived estimates were consistent (Supporting Information Table S9a‐S9c for whole sample, males, and females, respectively). The MR‐Egger intercept estimate showed some evidence for pleiotropy in the association between BMI and mortality from other cancers in the whole sample (Supporting Information Figure S2a) and males (Supporting Information Figure S2b), suggesting an underestimated MR estimate with negative directional pleiotropy (which was likely driven by the rs17024393 SNP). There was no strong evidence of directional pleiotropy in female‐specific analyses (Supporting Information Table S9c).

Additional adjustment for covariables made no substantive difference to the GRS‐BMI association (Supporting Information Table S10a) and MR analyses (Supporting Information Table S10b). When excluding genetic variants implicated as pleiotropic (*n* = 7; leaving 70 SNPs), there was no substantive difference in the GRS‐BMI association (Supporting Information Table S11a) and MR analyses (Supporting Information Table S11b).

## Discussion

Results supported the causal role of higher BMI in increasing the risk of all‐cause mortality and mortality specifically from CVDs plus various cancers, including esophageal cancer and stomach cancer, as well as decreasing lung cancer mortality risk. Sex‐stratified analyses were consistent with those in the whole sample and provided additional evidence for the causal role of higher BMI in increasing the risk of mortality from cancers of the kidney and liver in males and from external causes in females while decreasing the risk of mortality from bladder cancer in males and breast cancer (specifically postmenopausal breast cancer) and endometrial cancer in females.

The current results for the common mortality causes are consistent with previous studies 1, 2, 3, 4, 5, 10. For example, the largest systematic review and meta‐analysis of this relationship (including > 30 million participants and ~3.7 million deaths) showed consistent evidence that each 5 kg/m^2^ increment in BMI was associated with a 5% increased risk (95% CI: 4%‐7%) of all‐cause mortality 10. Concordant with this, scaling the current results in UK Biobank suggested that each 5 kg/m^2^ increase in BMI was associated with a ~16% increased all‐cause mortality risk (95% CI: −5% to 41%). Consistent with a collaborative analysis of > 900,000 adults showing a ~40% increased risk of vascular mortality with each 5‐kg/m^2^ higher BMI 1, scaling the current results to reflect the same increase in BMI implied a ~61% increased risk of overall CVD (HR 95% CI: 1.07‐2.43) and ~76% increased risk of CHD (HR 95% CI: 1.00‐3.11).

For cancer, many MR‐derived effect estimates were in the same direction as those derived from previous large‐scale meta‐analyses and reviews. For example, the association of BMI on incidence of 22 cancer sites in 5.24 million individuals suggested linear positive relationships with cancers of the kidney, liver, colorectal, and ovary and inverse associations with prostate, premenopausal breast cancer, and lung cancer, the latter being strongly driven by smoking status 11. Consistent with this, despite estimates from the Cox regression suggesting a positive association between BMI and prostate cancer mortality in UK Biobank, MR analyses provided evidence (with wide CIs) in the opposite direction (i.e., higher BMI reducing prostate risk). Additionally, in the Million Women Study, incrementally higher BMI was associated with an increased risk of mortality from cancers of the endometrium, esophagus, kidney, pancreas, lymphatic system, ovary, breast (in postmenopausal women), and colorectal (in premenopausal women) 3. While there was observational evidence for a positive association on mortality from endometrial cancer and postmenopausal breast cancer in the current study, estimates were inverse in MR analyses. However, analyses of cancer‐specific mortality in the current study were limited by the rarity of these deaths (i.e., many cancers had < 300 cases), which was accentuated further in sex‐stratified analyses in which many estimates derived from MR analyses were opposite of those from observational analyses or had CIs too wide for interpretation.

The association between BMI and all‐cause mortality in MR analyses showed a J‐shaped pattern but appeared flatter over a larger range of BMI compared with the observational association, with a smaller value of BMI at which mortality risk was lowest. This difference may be suggestive of confounding in previous observational associations, which overestimate the harmful effects of having underweight while underestimating the harmful effects of having overweight or obesity. For example, studies using populations comprising older individuals with likely existing illnesses can generate spurious associations between lower BMI and increased risk of mortality (i.e., those who lose weight because of disease) 9, 31, 41. Indeed, in the largest study to date, overestimation of estimates and this characteristic J‐shaped association were reported greatest in analyses with the most potential for bias (including all participants; current, former or never smokers; and studies with short follow‐up of < 5 years), highlighting the importance for unbiased modes of estimation (such as those used here) 10. Those that attempt to appropriately control for such effects (i.e., adjusting for baseline traits, restricting analyses to individuals who never smoked or had a longer follow‐up), observe an emerging linear association 2, 10, 42, 43. While it is plausible that individuals considered to have severe and unhealthy underweight have a higher risk of mortality than those within the normal BMI range 44, the current findings in this large population of healthy individuals support a more linear association, with lower BMI being protective over most of the observed range. Furthermore, the lowest risk of mortality occurred at approximately 23 kg/m^2^ with MR as opposed to being overweight (i.e., BMI 25.0‐29.9 kg/m^2^), which was observed in the current observational analyses and has been implied previously by some existing observational studies 7. Therefore, a stable BMI within the “normal” range (i.e., 18.5‐24.9 kg/m^2^) may be the most beneficially healthy in reducing mortality risk, with any reduction within that range likely to be favorable 5, 10.

The MR concept rests on several of the following key assumptions 14, 15: (1) the GRS must be associated with BMI, (2) the GRS must be independent of the confounding factors of the association between BMI and mortality, and (3) there must be no independent pathway between the GRS and mortality other than through BMI‐horizontal pleiotropy 15. These assumptions were tested where possible, and sensitivity analyses conducted in the current study provided little evidence of confounding or pleiotropy and awarded greater confidence in the validity of the instrument used and, thus, MR‐derived estimates. Notably, the GRS was associated (with very small effect sizes) with covariables. The sheer presence of an association between traditionally considered confounders with the GRS is interesting and could be because of (1) vertical pleiotropy (i.e., the GRS being associated with smoking status, for example, because of the potentially causal relationship between BMI and smoking) or (2) coincident genetic and phenotypic variation because of population structure or selection/collider bias; both reasons are increasingly easier to detect with the advent of very large studies such as UK Biobank 45. Nevertheless, the magnitude of these relationships was marginal, and MR analyses adjusting for these covariables were consistent with main analyses, suggesting little impact. Reverse causality is an important source of bias in observational estimates of the association between BMI and mortality and may be the driver of the characteristic J‐shaped association. While it is possible that mortality may influence the relative distribution of genetic variants within a selected sample 45, it is likely that this potential bias is less marked than that seen in observational studies. Though there are limitations to this current study, triangulation of different methodologies (each with orthogonal sources of bias) is important for drawing causal inference within this context, and these findings add to the current body of evidence aiming to estimate the role played by BMI in mortality.

The UK Biobank study is a unique opportunity to undertake these analyses; however, there are important aspects to consider. First, current analyses were restricted to those of White British ancestry, limiting the generalizability of results to other ancestral groups. Second, one cannot rule the coincident structure in both genotype and phenotype out of any potential biasing role in genetic analyses within a study of this scale. Lastly, the power to detect associations with MR analyses remains low for many mortality causes even in a study comprising ~500,000 participants. Despite these, and given the incidence of the outcomes tested (in which incidence of mortality from many causes will approximately double by 2022) 28, UK Biobank provides a unique opportunity to analyze and revise these estimates further over the coming years.

## Conclusion

This study represents the application of MR to assess the causal effect of higher BMI on the risk of mortality. Results supported the causal role of higher BMI in increasing the risk of all‐cause mortality and mortality from CVDs, various cancers, and several specific causes. Alongside more large‐scale comprehensive studies and the application of robust causal inference methods that appropriately account for the heavy burden of confounding, reverse causation, and bias within observational epidemiological designs, our results further highlight the need for a global effort to reduce the rising population trends for excess weight.

## Funding agencies

NJT is a Wellcome Trust Investigator (202802/Z/16/Z) and a work‐package lead in the Integrative Cancer Epidemiology Programme that is supported by a Cancer Research UK programme grant (C18281/A19169), and he works within the University of Bristol National Institute for Health Research Biomedical Research Centre. GDS is the director and a programme lead in the Medical Research Council Integrative Epidemiology Unit (MRC IEU) (grant codes for 2013‐2018: MC_UU_12013/1‐6 and for 2018‐2023: MC_UU_00011/1‐7). At the start of this project, KHW was funded equally by two programs of the MRC‐IEU (grant codes for 2013‐2018: MC_UU_12013/3 and MC_UU_12013/4) and is now funded by the Wellcome Trust Investigator award (202802/Z/16/Z, PI: NJT). At the start of this project, DC was funded by the statistics theme of the MRC‐IEU (grant code for 2013‐2018: MC_UU_12013/9) and is now affiliated to program 1 of the MRC‐IEU (grant code for 2013‐2018: MC_UU_12013/1 and for 2018‐2023: MC_UU_00011/1). The funders of the study had no role in the study design, data collection, data analysis, data interpretation, or writing of the report.

## Disclosure

NS is a member of the UK Biobank International Scientific Advisory Board and Enhancements Committee. This had no bearing on the study. The other authors declared no conflicts of interest.

## Author contributions

All authors conceived and designed the study, interpreted results, and approved the final manuscript. KHW had full access to all the data and performed all analyses with DC and takes responsibility for the integrity of the data and accuracy of the analysis. KHW, DC, and NJT drafted the initial manuscript, the final version of which was critically appraised, revised, and approved by all authors.
