# Supplementary material for: BMI and Mortality in UK Biobank: Revised Estimates Using Mendelian Randomization
Source: Obesity (Silver Spring). 2018 Oct 25;26(11):1796–806. doi: 10.1002/oby.22313 (PMC6334168; doi:10.1002/oby.22313)
Supplement: Supplementary file 1 [file OBY-26-1796-s001.docx]

**Supporting Information**

Body mass index and mortality in UK Biobank: revised estimates using Mendelian randomization

Kaitlin H Wade^1,2^, David Carslake^1,2^, Naveed Sattar^3^, George Davey Smith^1,2^, Nicholas J Timpson^1,2^

^1^MRC Integrative Epidemiology Unit, University of Bristol, Bristol, BS8 2BN

^2^Population Health Sciences, Bristol Medical School, Faculty of Health Sciences, University of Bristol, BS8 2BN

^3^Institute of Cardiovascular and Medical Sciences, British Heart Foundation Glasgow Cardiovascular Research Centre, University of Glasgow, Glasgow, G12 8TA

Corresponding author:

Dr Kaitlin H Wade

Oakfield House

Oakfield Road

Clifton

Bristol

BS8 2BN

Telephone: +44(0)1173314092

Email: [kaitlin.wade@bristol.ac.uk](mailto:kaitlin.wade@bristol.ac.uk)

**Supporting Methods**

*The UK Biobank Study*

The UK Biobank study recruited over 500,000 people aged 37-73 years (99.5% were between 40 and 69 years) from across the country in 2006-2010. Particularly focused on identifying determinants of human diseases in middle-aged and older individuals, participants provided a range of information (such as demographics, health status, lifestyle measures, cognitive testing, personality, self-report and physical/mental health measures) via questionnaires and interviews; anthropometric measures, BP readings and samples of blood, urine and saliva were taken. A full description of the study design, participants and quality control (QC) methods has been described in detail previously^1-3^. UK Biobank received ethical approval from the Research Ethics Committee (REC reference: 11/NW/0382).

Details of patient and public involvement in the UK Biobank are available online ([www.ukbiobank.ac.uk/about-biobank-uk/](http://www.ukbiobank.ac.uk/about-biobank-uk/) and <https://www.ukbiobank.ac.uk/wp-content/uploads/2011/07/Summary-EGF-consultation.pdf?phpMyAdmin=trmKQlYdjj-nQIgj%2C-fAzikMhEnx6>) and is available in a pre-print version^2^. No patients were specifically involved in setting the research question or the outcome measures, nor were they involved in developing plans for recruitment, design or implementation of this study. No patients were asked to advise on the interpretation or writing up of the results. There are no specific plans to disseminate the results of the research to study participants, but the UK Biobank disseminates key findings from the projects on its websites. At the time of this study, phenotypic data were available for 502,619 participants.

*Measures of body mass index*

Weight and height were collected at baseline when participants attended the initial assessment centre. Height (cm) was measured using a Seca 202 device in all participants in the UK Biobank along with sitting height. Weight (kg) was measured by a variety of means during the initial Assessment Centre visit, which was amalgamated into a single weight variable on the UK Biobank release data.

A total of 13 participants had a height measurement more than 4.56 standard deviations (SDs) away from the mean and one person had a sitting to standing height ratio of greater than 0.75, which is not compatible with normal growth and development^4^. These participants were excluded, leaving 500,066 valid height measurements. Of these, 499,504 participants had weight measurements available (no weight values were excluded).

The UK Biobank currently has two different measures of adiposity – body mass index (BMI) calculated as weight divided by height squared (kg/m^2^) measured at the initial Assessment Centre visit and mass quantified using electrical impedance (in increments of 0.1kg), which was used to calculate a second measure of BMI. If BMI measured at the initial Assessment Centre visit was not available, the electrical impedance measure was used (n=255). Participants with substantial differences (>4.56 SD^4^) between impedance and normal BMI measures were excluded (n=1,164), if both measures were available. After these preliminary steps, 498,595 participants had a valid BMI measurement (see Figure 1 in the main manuscript document for flow-chart of the participants used in this analysis).

*All-cause and cause-specific mortality*

Data from death certificates were sent to UK Biobank on a quarterly basis provided by the National Health Service (NHS) Information Centre for participants from England and Wales and by NHS Central Register, Scotland for participants from Scotland. More detailed information on mortality are available at <http://biobank.ctsu.ox.ac.uk/crystal/refer.cgi?id=115559>. The death certificates include the disease or condition stated to be the underlying cause of death, as well as other conditions, diseases, injuries or events contributing to death but not related to the disease or condition causing it. Data were provided as date of death (DoD), an integer value for age of death (AoD) and underlying (primary) cause of death in International Classification of Diseases (ICD)-10 codes for all deaths that occurred between the 10/05/2006 and 16/02/2016. Rather than using the integer value of AoD from the death certificate, a more precise measure of AoD was derived by adding the time interval between date of initial assessment and DoD (in days) to the participant’s age at initial assessment. All participants who were not recorded as dead by the 16^th^ of February 2016 were assumed to still be alive. The ICD-10 codes were categorised into all-cause and cause-specific mortality as presented in Table S1a. As of August 2017 (date of extraction for all data), there were 14,417 total deaths in the entire UK Biobank dataset that had occurred up to 16^th^ of February 2016 (Table S1a for the whole sample and Table S1b for males and females), which remains the most updated data on mortality.

For the purposes of this study, the primary outcomes of focus were as follows: all-cause mortality and mortality from all cardiovascular diseases and those specifically due to coronary heart disease, stroke, aortic aneurysm and any other cardiovascular diseases; overall cancer and those specifically due to cancers of the lung, colorectum, prostate (men only), breast cancer (women only, separated into pre- and post-menopausal occurrences), pancreas, ovaries (women only), endometrium (women only), stomach, oesophagus, skin (malignant melanoma), kidney, bladder, brain, lymphatic system and all other cancers; and external causes. All other causes were combined, analysed separately and presented in Supporting Information.

*Covariables*

At the initial UK Biobank Assessment Centre, participants were given a touchscreen questionnaire, which included questions about sociodemographic status, early life, sex-specific factors, lifestyle and environment, family history, health and medical history and psychosocial factors. Of the sociodemographic questions, participants were asked whether they had any of the following qualifications or equivalent: i) college or university degree, ii) A/AS-levels, iii) O-levels/GCSEs, iv) CSEs, v) NVQ or HND or HNC, vi) other professional qualifications (e.g., nursing or teaching), vii) none of the listed. Additionally, participants were asked which of the following described their current employment situation: i) in paid employment or self-employed, ii) retired, iii) looking after family home and/or family, iv) unable to work because of sickness or disability, v) unemployed, vi) doing unpaid or voluntary work, vii) full or part-time student, viii) none of the listed. Answers to these questions were used to derive variables the represented the participants’ highest qualification level and current employment status, respectively.

Of the lifestyle and environment questions, participants were asked their smoking and alcohol drinking status, categorised into ‘never’, ‘former or ‘current’. Participants were also asked how many days in a typical week they would do 10 or more minutes of vigorous physical activity (“activities that make you sweat or breathe hard such as fast cycling, aerobic exercise and heavy lifting”).

*Genotyping*

Pre-imputation, QC, phasing and imputation of UK Biobank have been described elsewhere^2, 5^. The genetic variants used were extracted genotypes from the UK Biobank imputation dataset (using only genetic variants imputed to the Haplotype Reference Consortium (HRC) reference panel), which had extensive QC performed including exclusion of the majority of third degree or closer relatives from a genetic kinship analysis of 96% of participants. For more details, see <http://biobank.ctsu.ox.ac.uk>. A total of 77 common genetic variants associated with BMI within people of only European ancestry (and excluding those that reached genome-wide levels of statistical confidence in only one sex or one stratum) in the most updated genome-wide association study (GWAS) conducted by the Genetic Investigation of ANthropometric Traits (GIANT) consortium (comprising up to 339,224 people) were extracted for MR analyses (Table S2)^4, 6^. One SNP from the GWAS (rs12016871) was not present in the UK Biobank imputed genetic data, so a proxy SNP (i.e., one that is in linkage disequilibrium [LD] with rs12016871) was identified (rs4771122; r^2^=0.876, distance=2398bp) and used in replacement^6^. Each of the variants was imputed with high quality (>0.90, Table S2).

The dosage of each genetic variant was weighted by its relative effect size on BMI obtained from the GIANT consortium^6^ and summed across all variants. The resulting total was then rescaled by dividing by the sum of all effect sizes on BMI obtained from the GIANT consortium^6^ and multiplied by the number of genetic variants used. Therefore, this weighted genetic risk score (GRS) reflected the number of average BMI-increasing alleles each participant possessed^4^. In total, 487,409 participants had genetic data.

*Standard exclusions*

After preliminary exclusions, 484,514 individuals had a valid measure of BMI, plausible age and death data, along with available genetic data (see Figure 1 of main manuscript document). The following exclusions were made to the dataset required for survival analyses prior to all analyses based on in-house QC parameters (total excluded = 149,206; see Figure S1 below). Individuals who were outliers in heterozygosity and missing rates (f.22027) were already excluded from imputed data.

- Sex mismatch (N=367) – derived by comparing the genetic sex variable (f.22001), as determined by Affymetrix, with reported sex (f.31) of the participant.
- Sex-chromosome aneuploidy (N=643) – individuals with sex chromosome karyotypes putatively different from XX or XY (f.22019). Of these, 177 individuals overlapped with the sex-mismatch list (above).
- Relatedness – minimally related individuals were removed (N=79,034), which were defined as the first individual in a related pair (3^rd^ degree or closer) based on an algorithm applied to the list of all the related pairs provided by UK Biobank. This number also included any individuals who appeared to be highly related to a very large number (>200) of individuals, derived using the list of individuals excluded from the kinship inference (N=9). Of these, 106 individuals overlapped with those excluded based on sex mismatch and sex-chromosome aneuploidy (above). Once removed, the remaining subset was the maximal set of unrelated individuals in UK Biobank.
- Ancestry – stringent criteria for excluding those not of White British ancestry was used, retaining those who self-reported as “White” and “British” and had very similar genetic ancestry based on a principal components analysis of the genotypes (N=77,722). Of these, 8,277 individuals overlapped with those excluded based on sex mismatch, sex-chromosome aneuploidy and relatedness (above).

Therefore, of those with full genetic data and information on BMI, 335,308 participants of White British ancestry were included in analyses after recommended exclusions based on sex mismatch, sex-chromosome aneuploidy detection and related individuals. Of these, 9,570 had available data on cause, age and date of death (see Table S1a and Figure 1 in the main manuscript).

*Statistical analysis*

A total of 77 common genetic variants associated with BMI within people of only European ancestry (and excluding those that reached genome-wide levels of statistical confidence in only one sex or one stratum) in the most updated genome-wide association study (GWAS) conducted by the Genetic Investigation of ANthropometric Traits (GIANT) consortium were extracted for MR analyses (Table S2)^4, 6^. The dosage of each genetic variant was weighted by its relative effect size on BMI in the GIANT consortium^6^ and summed across all variants. The resulting total was then rescaled such that the weighted genetic risk score (GRS) reflected the number of average BMI-increasing alleles each participant possessed^4^.

As only the month and year of birth was available in the UK Biobank study, date of birth (DoB) was set as the 15^th^ of each month and year in which the participant was born. Participants were removed if they lacked information on date of birth (used for secular trends), initial assessment age and date, cause of death or AoD. Participants were also excluded if they lacked any/plausible information on DoD (i.e., if the individual had apparently died before the assessment clinic that they later attended). Participants who were never at risk during the follow-up period (i.e., who were recruited after 16^th^ February 2016) were also excluded.

*Test for endogeneity between conventional Cox regression and MR analyses*

A simplification of the matrix method for the Durbin-Wu-Hausman (DWH) test for endogeneity was used to compare effect estimates derived from conventional Cox regression and Mendelian randomization (MR) analyses. For one instrumental variable (here, a weighted genetic risk score [GRS]), the test statistic can be simplified to the following formula:

$$H= \frac{{(\beta_{CCR}-\beta_{MR})}^{2}}{({{SE}_{CCR}}^{2}-{{SE}_{MR}}^{2})}$$

where $\beta_{CCR}$ and $\beta_{MR}$ are the effect estimates obtained from the conventional Cox regression and MR analyses, respectively, and ${SE}_{CCR}$ and ${SE}_{MR}$ are the corresponding standard errors. The test statistic, $H$, has a chi-squared distribution with one degree of freedom^7^.

*Sensitivity analysis*

To investigate the validity of the GRS as an IV within this context, MR-Egger was used to detect and accommodate violations of the MR assumptions, specifically horizontal pleiotropy^8^. The intercept obtained from the MR-Egger test is used as an indication of pleiotropy and the slope can be considered as the estimate of the causal effect between the exposure (here, BMI) and the outcome (here, all-cause and cause-specific mortality). In addition, the weighted median- and mode-based methods were used^9^, which vary in their assumptions of instrument validity. The weighted median approach provides a causal estimate even when 50% of instruments are invalid and the weighted mode estimate is consistent when the largest number of similar causal effect estimates comes from valid instruments, even if most instruments are invalid. The MR-Egger, weighted median and weighted mode estimates were compared to those obtained from the inverse-variance weighted (IVW) method for two-sample MR^8, 10^. For these analyses, the first-stage estimates (coefficients of the association between each SNP and BMI) were obtained from an independent external source, as to not induce weak instrument bias^11, 12^, and the second-stage estimates (natural logarithm of the HR for each mortality outcome with each SNP, , adjusted for secular trend and the first ten genetic principal components) were obtained directly from UK Biobank.

In the UK Biobank sample, there is evidence to suggest a differential array effect on markers scattered across the genome (i.e., those that were genotyped using the Affymetrix UK Biobank Axiom® Array or the Affymetrix UK BiLEVE Axiom Array^2, 5^) and the UK BiLEVE sub-sample, which included >50,000 participants and used the UK BiLEVE Axiom Array, also preferentially selected individuals based on smoking intensity. To evaluate the impact of this differential array effect, and the confounding factors associated with the GRS, MR sensitivity analyses were conducted with adjustment for these additional covariables.

As a final sensitivity analysis, the GRS was restricted to exclude the genetic variants known to be classified as having a secondary signal within a locus to other phenotypes (N=7; leaving 70 in the GRS, Table S3)^4, 13^ and all MR analyses were repeated.

Table S1a. Descriptive statistics for UK Biobank mortality data

| **Cause of death** | **ICD10 codes** | **Total number of deaths in UK Biobank** | | |
| --- | --- | --- | --- | --- |
|  |  | **Whole sample** | **With valid data** | **White British** |
| All-cause | All | 14,417 | 11,161 | 9,570 |
| Cardiovascular disease*^1^* | I*; G459 | 2,999 | 2,332 | 1,967 |
| Coronary heart disease*^2^* | I209-I259; I516 | 1,661 | 1,291 | 1,087 |
| Stroke*^3^* | I600-I698; G459 | 550 | 428 | 346 |
| Aortic aneurysm | I710-I719 | 152 | 120 | 109 |
| Other cardiovascular diseases | All other I | 636 | 493 | 425 |
| **Diabetes*^4^*** | **E10-E149** | **70** | **50** | **37** |
| Respiratory diseases*^5^* | J* | 834 | 631 | 532 |
| Cancer^6^ | C* | 8,286 | 6,484 | 5,613 |
| Lung cancer | C33-C349 | 1,507 | 1,160 | 993 |
| Breast cancer | C50-C509 | 739 | 560 | 472 |
| Prostate cancer | C61-C619 | 440 | 348 | 308 |
| Colorectal cancer | C180-C219 | 822 | 646 | 552 |
| Pancreatic cancer | C250-C259 | 556 | 446 | 388 |
| Stomach cancer | C160-C169 | 201 | 162 | 144 |
| Ovarian cancer | C56-C570 | 306 | 247 | 211 |
| Endometrial cancer | C54-C549 | 76 | 56 | 50 |
| **Gallbladder cancer** | **C23-C249** | **55** | **41** | **37** |
| Oesophageal cancer | C15-C159 | 389 | 310 | 283 |
| Malignant melanoma | C43-C449 | 173 | 131 | 119 |
| **Thyroid cancer** | **C73-C739** | **16** | **11** | **9** |
| Kidney cancer | C64-C669 | 245 | 196 | 181 |
| Bladder cancer | C67-C679 | 161 | 122 | 101 |
| Brain cancer | C71-C729 | 408 | 322 | 280 |
| Liver cancer | C220-C229 | 266 | 208 | 169 |
| **Cervical cancer** | **C53-C539** | **16** | **13** | **12** |
| **Uterine cancer** | **C55-C559** | **40** | **29** | **21** |
| Lymphatic cancer | C810-C964 | 730 | 592 | 528 |
| Other cancers | All other C | 1,140 | 884 | 755 |
| **Kidney disease** | **N00-N299** | **34** | **23** | **16** |
| **External causes*^7^*** | **V*; W*; X*; Y*** | **496** | **359** | **306** |
| Other causes*^8^* | - | 1,698 | 1,282 | 1,099 |

*ICD = International Classification of Diseases*

*Mortality causes in bold were not included in analyses due to small number of deaths (<40): cancer of the gallbladder (N=37), thyroid (N=9), cervix (N=12) and uterus (N=21) and kidney disease (N=16)*

**Any code beginning with the indicated letter*

*^1^Cardiovascular disease consisted of all disease of the circulatory system listed in ICD 10, including coronary heart disease and stroke.*

*^2^Coronary heart disease is a narrowing of the arteries supplying the heart muscle and may be considered synonymous with ischemic heart disease or coronary artery disease.*

*^3^Stroke included bleeding from (haemorrhagic stroke) or blockage of (ischemic stroke) the arteries supplying the brain, as well as transient ischemic attacks (“mini-strokes”).*

*^4^Diabetes included insulin-dependent and non-insulin-dependent diabetes mellitus.*

*^5^Respiratory diseases included all non-neoplastic diseases of the lungs, pleura and respiratory tract.*

*^6^Cancers excluded benign or in-situ neoplasms.*

*^7^External causes consisted of accidents and violence, including suicide and conditions consequent to accidents and violence*

*^8^Other causes included all other causes of mortality not otherwise listed.*

Table S1b. Descriptive statistics for UK Biobank mortality data stratified by sex

| **Cause of death** | **ICD10 codes** | **Total number of deaths in UK Biobank (with valid data for main analyses)** | |
| --- | --- | --- | --- |
|  |  | **Males** | **Females** |
| All-cause | All | 5,882 | 3,688 |
| Cardiovascular disease*^1^* | I*; G459 | 1,467 | 500 |
| Coronary heart disease*^2^* | I209-I259; I516 | 906 | 181 |
| Stroke*^3^* | I600-I698; G459 | 194 | 152 |
| **Aortic aneurysm** | **I710-I719** | 83 | **26** |
| Other cardiovascular diseases | All other I | 284 | 141 |
| **Diabetes*^4^*** | **E10-E149** | **29** | **8** |
| Respiratory diseases*^5^* | J* | 361 | 171 |
| Cancer^6^ | C* | 3,113 | 2,500 |
| Lung cancer | C33-C349 | 571 | 422 |
| **Breast cancer** | **C50-C509** | **4** | 468 |
| Prostate cancer | C61-C619 | 308 | - |
| Colorectal cancer | C180-C219 | 329 | 223 |
| Pancreatic cancer | C250-C259 | 201 | 187 |
| **Stomach cancer** | **C160-C169** | 105 | **39** |
| Ovarian cancer | C56-C570 | - | 211 |
| Endometrial cancer | C54-C549 | - | 50 |
| **Gallbladder cancer** | **C23-C249** | **16** | **21** |
| Oesophageal cancer | C15-C159 | 226 | 57 |
| Malignant melanoma | C43-C449 | 78 | 41 |
| **Thyroid cancer** | **C73-C739** | **2** | **7** |
| Kidney cancer | C64-C669 | 137 | 44 |
| **Bladder cancer** | **C67-C679** | 78 | **23** |
| Brain cancer | C71-C729 | 169 | 111 |
| Liver cancer | C220-C229 | 100 | 69 |
| **Cervical cancer** | **C53-C539** | **-** | **12** |
| **Uterine cancer** | **C55-C559** | **-** | **21** |
| Lymphatic cancer | C810-C964 | 329 | 199 |
| Other cancers | All other C | 460 | 295 |
| **Kidney disease** | **N00-N299** | **10** | **6** |
| External causes*^7^* | V*; W*; X*; Y* | 206 | 100 |
| Other*^8^* | - | 696 | 403 |

*ICD = International Classification of Diseases*

*In addition to the mortality causes with a collectively small number of deaths in the whole sample (Table S1a), the sex-specific mortality causes in bold were also not included in analyses due to small numbers of deaths (<40) when stratified by sex: aortic aneurysm in females (N=26), diabetes (N=29/8 in males/females, respectively), breast cancer in males (N=4) and cancer of the stomach in females (N=39), gallbladder (N=16/21 in males/females, respectively) and bladder in females (N=23).*

**Any code beginning with the indicated letter*

*^1^Cardiovascular disease consisted of all disease of the circulatory system listed in ICD 10, including coronary heart disease and stroke.*

*^2^Coronary heart disease is a narrowing of the arteries supplying the heart muscle and may be considered synonymous with ischemic heart disease or coronary artery disease.*

*^3^Stroke included bleeding from (haemorrhagic stroke) or blockage of (ischemic stroke) the arteries supplying the brain, as well as transient ischemic attacks (“mini-strokes”).*

*^4^Diabetes included insulin-dependent and non-insulin-dependent diabetes mellitus.*

*^5^Respiratory diseases included all non-neoplastic diseases of the lungs, pleura and respiratory tract.*

*^6^Cancers excluded benign or in-situ neoplasms.*

*^7^External causes consisted of accidents and violence, including suicide and conditions consequent to accidents and violence*

*^8^Other causes included all other causes of mortality not otherwise listed.*

Table S2. Genetic variants (N=77) associated with BMI in the GIANT consortium and available in UK Biobank individuals (N=335,308)

| **Genetic variant** | **Gene** | **Chr** | **bp** | **Effect allele^1^** | **Other allele^1^** | **EAF^2^** | **Beta (SE)^3^** | **P-value^3^** | **Imputation quality^2^** |
| --- | --- | --- | --- | --- | --- | --- | --- | --- | --- |
| rs1000940 | *RABEP1* | 17 | 5283252 | G | A | 0.30 | 0.07 (0.01) | 1.45x10^-07^ | 0.998 |
| rs10132280 | *STXBP6* | 14 | 25928179 | C | A | 0.70 | 0.11 (0.01) | 2.54x10^-17^ | 0.988 |
| rs1016287 | *LINC01122* | 2 | 59305625 | T | C | 0.30 | 0.10 (0.01) | 1.04x10^-14^ | 0.997 |
| rs10182181 | *ADCY3* | 2 | 25150296 | G | A | 0.49 | 0.17 (0.01) | 1.42x10^-48^ | 0.996 |
| rs10733682 | *LMX1B* | 9 | 129460914 | A | G | 0.47 | 0.06 (0.01) | 1.63x10^-07^ | 0.962 |
| rs10938397 | *GNPDA2* | 4 | 45182527 | G | A | 0.43 | 0.14 (0.01) | 9.04x10^-34^ | 1.000 |
| rs10968576 | *LINGO2* | 9 | 28414339 | G | A | 0.31 | 0.12 (0.01) | 7.92x10^-23^ | 1.000 |
| rs11030104 | *BDNF* | 11 | 27684517 | A | G | 0.80 | 0.18 (0.01) | 1.77x10^-35^ | 0.999 |
| rs11057405 | *CLIP1* | 12 | 122781897 | G | A | 0.90 | 0.14 (0.02) | 3.10x10^-14^ | 1.000 |
| rs11126666 | *KCNK3* | 2 | 26928811 | A | G | 0.26 | 0.02 (0.01) | 0.16 | 0.995 |
| rs11165643 | *PTBP2* | 1 | 96924097 | T | C | 0.58 | 0.08 (0.01) | 9.47x10^-13^ | 0.998 |
| rs11191560 | *NT5C2* | 10 | 104869038 | C | T | 0.08 | 0.12 (0.02) | 1.42x10^-08^ | 0.999 |
| rs11583200 | *ELAVL4* | 1 | 50559820 | C | T | 0.40 | 0.07 (0.01) | 1.78x10^-09^ | 0.993 |
| rs1167827 | *HIP1* | 7 | 75163169 | G | A | 0.57 | 0.11 (0.01) | 6.81x10^-20^ | 1.000 |
| rs11688816 | *EHBP1* | 2 | 63053048 | G | A | 0.54 | 0.06 (0.01) | 1.76x10^-07^ | 0.993 |
| rs11727676 | *HHIP* | 4 | 145659064 | T | C | 0.91 | 0.04 (0.02) | 0.03 | 1.000 |
| rs11847697 | *PRKD1* | 14 | 30515112 | T | C | 0.05 | 0.12 (0.03) | 3.99x10^-05^ | 1.000 |
| rs12286929 | *CADM1* | 11 | 115022404 | G | A | 0.52 | 0.08 (0.01) | 9.79x10^-11^ | 0.996 |
| rs12401738 | *FUBP1* | 1 | 78446761 | A | G | 0.33 | 0.07 (0.01) | 4.54x10^-10^ | 0.995 |
| rs12429545 | *OLFM4* | 13 | 54102206 | A | G | 0.13 | 0.13 (0.02) | 3.38x10^-13^ | 0.979 |
| rs12446632 | *GPRC5B* | 16 | 19935389 | G | A | 0.86 | 0.13 (0.02) | 1.77x10^-15^ | 1.000 |
| rs12566985 | *FPGT-TNNI3K* | 1 | 75002193 | G | A | 0.45 | 0.07 (0.01) | 1.14x10^-09^ | 0.998 |
| rs12885454 | *PRKD1* | 14 | 29736838 | C | A | 0.65 | 0.07 (0.01) | 1.82x10^-09^ | 0.998 |
| rs12940622 | *RPTOR* | 17 | 78615571 | G | A | 0.56 | 0.08 (0.01) | 2.83x10^-12^ | 0.999 |
| rs13021737 | *TMEM18* | 2 | 632348 | G | A | 0.83 | 0.25 (0.02) | 6.91x10^-59^ | 1.000 |
| rs13078960 | *CADM2* | 3 | 85807590 | G | T | 0.20 | 0.10 (0.01) | 4.96x10^-11^ | 0.994 |
| rs13107325 | *SLC39A8* | 4 | 103188709 | T | C | 0.07 | 0.25 (0.02) | 2.82x10^-29^ | 1.000 |
| rs13191362 | *PARK2* | 6 | 163033350 | A | G | 0.88 | 0.10 (0.02) | 7.79x10^-09^ | 0.995 |
| rs1516725 | *ETV5* | 3 | 185824004 | C | T | 0.86 | 0.16 (0.02) | 6.64x10^-22^ | 0.992 |
| rs1528435 | *UBE2E3* | 2 | 181550962 | T | C | 0.62 | 0.08 (0.01) | 4.57x10^-11^ | 0.997 |
| rs1558902 | *FTO* | 16 | 53803574 | A | T | 0.39 | 0.36 (0.01) | 5.55x10^-201^ | 1.000 |
| rs16851483 | *RASA2* | 3 | 141275436 | T | G | 0.07 | 0.17 (0.02) | 3.36x10^-13^ | 0.998 |
| rs16951275 | *MAP2K5* | 15 | 68077168 | T | C | 0.77 | 0.14 (0.01) | 9.63x10^-23^ | 0.999 |
| rs17001654 | *SCARB2* | 4 | 77129568 | G | C | 0.15 | 0.09 (0.02) | 2.65x10^-07^ | 0.973 |
| rs17024393 | *GNAT2* | 1 | 110154688 | C | T | 0.03 | 0.31 (0.04) | 1.15x10^-17^ | 0.998 |
| rs17094222 | *HIF1AN* | 10 | 102395440 | C | T | 0.21 | 0.07 (0.01) | 4.01x10^-07^ | 0.991 |
| rs17405819 | *HNF4G* | 8 | 76806584 | T | C | 0.71 | 0.09 (0.01) | 9.08x10^-14^ | 1.000 |
| rs17724992 | *PGPEP1* | 19 | 18454825 | A | G | 0.73 | 0.08 (0.01) | 2.93x10^-09^ | 0.991 |
| rs1808579 | *C18orf8* | 18 | 21104888 | C | T | 0.52 | 0.10 (0.01) | 3.91x10^-18^ | 0.998 |
| rs1928295 | *TLR4* | 9 | 120378483 | T | C | 0.57 | 0.06 (0.01) | 1.49x10^-06^ | 1.000 |
| rs2033529 | *TDRG1* | 6 | 40348653 | G | A | 0.28 | 0.11 (0.01) | 3.39x10^-17^ | 0.992 |
| rs2033732 | *RALYL* | 8 | 85079709 | C | T | 0.75 | 0.05 (0.01) | 9.75x10^-05^ | 1.000 |
| rs205262 | *C6orf106* | 6 | 34563164 | G | A | 0.27 | 0.14 (0.01) | 1.36x10^-27^ | 0.998 |
| rs2075650 | *TOMM40* | 19 | 45395619 | A | G | 0.85 | 0.09 (0.02) | 1.39x10^-07^ | 1.000 |
| rs2112347 | *POC5* | 5 | 75015242 | T | G | 0.63 | 0.14 (0.01) | 2.21x10^-29^ | 1.000 |
| rs2121279 | *LRP1B* | 2 | 143043285 | T | C | 0.12 | 0.06 (0.02) | 0.002 | 0.991 |
| rs2176598 | *HSD17B12* | 11 | 43864278 | T | C | 0.25 | 0.09 (0.01) | 4.92x10^-12^ | 1.000 |
| rs2207139 | *TFAP2B* | 6 | 50845490 | G | A | 0.17 | 0.20 (0.02) | 9.44x10^-37^ | 1.000 |
| rs2245368 | *PMS2L11* | 7 | 76608143 | C | T | 0.18 | 0.11 (0.02) | 1.30x10^-13^ | 1.000 |
| rs2287019 | *QPCTL* | 19 | 46202172 | C | T | 0.82 | 0.15 (0.02) | 1.36x10^-24^ | 0.984 |
| rs2365389 | *FHIT* | 3 | 61236462 | C | T | 0.58 | 0.08 (0.01) | 2.94x10^-11^ | 0.994 |
| rs2650492 | *SBK1* | 16 | 28333411 | A | G | 0.29 | 0.09 (0.01) | 8.10x10^-12^ | 0.988 |
| rs2820292 | *NAV1* | 1 | 201784287 | C | A | 0.56 | 0.10 (0.01) | 9.50x10^-17^ | 1.000 |
| rs29941 | *KCTD15* | 19 | 34309532 | G | A | 0.67 | 0.08 (0.01) | 9.01x10^-10^ | 1.000 |
| rs3101336 | *NEGR1* | 1 | 72751185 | C | T | 0.61 | 0.10 (0.01) | 1.10x10^-18^ | 1.000 |
| rs3736485 | *DMXL2* | 15 | 51748610 | A | G | 0.47 | 0.06 (0.01) | 6.19x10^-07^ | 0.995 |
| rs3810291 | *ZC3H4* | 19 | 47569003 | A | G | 0.66 | 0.13 (0.01) | 2.87x10^-26^ | 1.000 |
| rs3817334 | *MTCH2* | 11 | 47650993 | T | C | 0.40 | 0.12 (0.01) | 8.11x10^-23^ | 1.000 |
| rs3849570 | *GBE1* | 3 | 81792112 | A | C | 0.35 | 0.06 (0.01) | 2.36x10^-06^ | 1.000 |
| rs3888190 | *ATP2A1* | 16 | 28889486 | A | C | 0.39 | 0.13 (0.01) | 7.39x10^-28^ | 1.000 |
| rs4256980 | *TRIM66* | 11 | 8673939 | G | C | 0.65 | 0.08 (0.01) | 8.96x10^-12^ | 0.996 |
| rs4740619 | *C9orf93* | 9 | 15634326 | T | C | 0.55 | 0.09 (0.01) | 3.62x10^-14^ | 0.999 |
| rs4771122* | *MTIF3* | 13 | 28020180 | G | A | 0.22 | 0.04 (0.01) | 0.01 | 1.000 |
| rs543874 | *SEC16B* | 1 | 177889480 | G | A | 0.20 | 0.23 (0.01) | 2.11x10^-58^ | 1.000 |
| rs6477694 | *EPB41L4B* | 9 | 111932342 | C | T | 0.36 | 0.06 (0.01) | 1.80x10^-06^ | 0.990 |
| rs6567160 | *MC4R* | 18 | 57829135 | C | T | 0.23 | 0.25 (0.01) | 6.70x10^-75^ | 0.998 |
| rs657452 | *AGBL4* | 1 | 49589847 | A | G | 0.40 | 0.08 (0.01) | 1.88x10^-11^ | 0.987 |
| rs6804842 | *RARB* | 3 | 25106437 | G | A | 0.57 | 0.05 (0.01) | 4.39x10^-06^ | 0.991 |
| rs7138803 | *BCDIN3D* | 12 | 50247468 | A | G | 0.36 | 0.13 (0.01) | 1.95x10^-28^ | 1.000 |
| rs7141420 | *NRXN3* | 14 | 79899454 | T | C | 0.52 | 0.10 (0.01) | 1.05x10^-17^ | 0.985 |
| rs7243357 | *GRP* | 18 | 56883319 | T | G | 0.82 | 0.09 (0.02) | 9.42x10^-09^ | 0.990 |
| rs758747 | *NLRC3* | 16 | 3627358 | T | C | 0.29 | 0.06 (0.01) | 9.10x10^-06^ | 0.978 |
| rs7599312 | *ERBB4* | 2 | 213413231 | G | A | 0.73 | 0.08 (0.01) | 7.13x10^-09^ | 0.979 |
| rs7899106 | *GRID1* | 10 | 87410904 | G | A | 0.05 | 0.13 (0.03) | 1.68x10^-06^ | 0.987 |
| rs7903146 | *TCF7L2* | 10 | 114758349 | C | T | 0.71 | 0.08 (0.01) | 1.04x10^-10^ | 1.000 |
| rs9400239 | *FOXO3* | 6 | 108977663 | C | T | 0.69 | 0.08 (0.01) | 3.48x10^-10^ | 0.994 |
| rs9925964 | *KAT8* | 16 | 31129895 | A | G | 0.65 | 0.12 (0.01) | 1.97x10^-24^ | 0.998 |

*BMI = body mass index; bp = base pair; Chr = chromosome; eaf = effect allele frequency; GIANT = Genetic Investigation of ANthropometric Traits; se = standard error*

*^1^Effect and other alleles associated with an increasing BMI, according to the most recent genome-wide association study of BMI^6^*

*^2^Minor allele frequency (MAF) of each genetic variant in UK Biobank and corresponding imputation quality (the latter of which was based on the whole of UK Biobank)*

*^3^Beta and corresponding standard error (SE) and P-value represent the change in BMI (kg/m^2^) per BMI-increasing allele of each genetic variant in individuals of White British ancestry adjusted for the first ten genetic principal components.*

**The rs4771122 SNP served as the closest proxy (with an r^2^=0.876, distance=2398bp), according to SNAP (*[*http://archive.broadinstitute.org/mpg/snap/ldsearch.php*](http://archive.broadinstitute.org/mpg/snap/ldsearch.php)*) for the rs12016871 on chromosome 13, which was not available in the UK Biobank genetic data.*

Table S3. Genetic variants excluded in from sensitivity analyses

| **SNP** | **Gene** | **Chromosome** | **Reason for exclusion** |
| --- | --- | --- | --- |
| rs977747 | *TAL1* | 1 | All ancestries |
| rs1460676 | *FIGN* | 2 | All ancestries |
| rs17203016 | *CREB1* | 2 | All ancestries |
| rs2176040 | *LOC646736* | 2 | European men |
| rs492400 | *USP37* | 2 | European men |
| rs13107325 | *SLC39A8* | 4 | Pleiotropic effects |
| rs17001654 | *SCARB2* | 4 | Pleiotropic effects |
| rs7715256 | *GALNT10* | 5 | All ancestries |
| rs13201877 | *IFNGR1* | 6 | All ancestries |
| rs9374842 | *LOC285762* | 6 | European population based |
| rs1167827 | *HIP1* | 7 | Pleiotropic effects |
| rs6465468 | *ASB4* | 7 | European women |
| rs9641123 | *CALCR* | 7 | European population based |
| rs16907751 | *ZBTB10* | 8 | European men |
| rs7903146 | *TCF7L2* | 10 | Identified in Corbin *et al*.^13^ |
| rs11030104 | *BDNF* | 11 | Pleiotropic effects |
| rs1441264 | *MIR548A2* | 13 | All ancestries |
| rs9540493 | *MIR548X2* | 13 | European population based |
| rs7164727 | *LOC100287559* | 15 | All ancestries |
| rs2080454 | *CBLN1* | 16 | All ancestries |
| rs3888190 | *ATP2A1* | 16 | Pleiotropic effects |
| rs4787491 | *INO80E* | 16 | European population based |
| rs9914578 | *SMG6* | 17 | All ancestries |
| rs7239883 | *LOC284260* | 18 | European women |
| rs2075650 | *TOMM40* | 19 | Pleiotropic effects |
| rs6091540 | *ZFP64* | 20 | European women |
| rs2836754 | *ETS2* | 21 | All ancestries |

*Consistent with previous studies^4, 13, 14^, these genetic variants were excluded in sensitivity analyses to test the robustness of the GRS used in MR analyses*

Table S4. Association of participants’ characteristics with BMI

| **Variable** | **N** | **Estimate (95% CI)^1^** | ***P*-value** |
| --- | --- | --- | --- |
| Age (years) | 335,308 | 0.03 (0.03, 0.03) | 1.15x10^-171^ |
| Sex (% of males) | 335,308 | 0.81 (0.78, 0.84) | <1.20x10^-307^ |
| *Smoking status* | 334,142 |  |  |
| Never |  | reference |  |
| Former |  | 0.81 (0.78, 0.85) | <1.20x10^-307^ |
| Current |  | -0.04 (-0.10, 0.01) | 0.11 |
| *Alcohol drinker status* | 335,074 |  |  |
| Never |  | reference |  |
| Former |  | 0.29 (0.16, 0.41) | 8.86x10^-06^ |
| Current |  | -0.68 (-0.77, -0.59) | 9.22x10^-47^ |
| *Highest qualifications* | 275,544 |  |  |
| College or University degree |  | reference |  |
| A-levels |  | 0.59 (0.54, 0.64) | 5.98x10^-101^ |
| O-levels |  | 1.00 (0.96, 1.05) | <1.20x10^-307^ |
| CSEs |  | 1.49 (1.42, 1.56) | <1.20x10^-307^ |
| NVQ/HND/HNC |  | 1.71 (1.64, 1.78) | <1.20x10^-307^ |
| Other professional qualifications |  | 1.17 (1.09, 1.24) | 4.16x10^-205^ |
| *Current employment status* | 332,835 |  |  |
| In paid employment or self-employed |  | reference |  |
| Retired |  | 0.27 (0.24, 0.31) | 4.55x10^-55^ |
| Looking after home/family |  | -0.70 (-0.80, -0.60) | 7.82x10^-42^ |
| Unable to work due to sickness/disability |  | 2.50 (2.40, 2.59) | <1.20x10^-307^ |
| Unemployed |  | 0.84 (0.70, 0.98) | 1.77x10^-31^ |
| Doing unpaid or voluntary work |  | -0.74 (-0.99, -0.49) | 5.50x10^-09^ |
| Full or part-time student |  | -0.75 (-1.13, -0.38) | 6.94x10^-05^ |
| Days/week spent doing vigorous physical activity | 319,813 | -0.24 (-0.24, -0.23) | <1.20x10^-307^ |
| Genotyping chip^2^ | 335,308 | 0.61 (0.55, 0.66) | 7.02x10^-102^ |

*BMI = body mass index; CI = confidence interval; CSE = certificate of secondary education; HNC = higher national certificate; HND = higher national diploma; NVQ = national vocational qualification*

*^1^Estimates represent the difference in BMI (kg/m^2^) per unit increase in each continuous, categorical or binary variable in individuals of White British ancestry.*

*^2^There was evidence of differential array effect on markers scattered across the genome; therefore, the UK BiLEVE study genotyped on the Affymetrix Axiom Array was considered as a covariable*

Table S5. Association of participants’ characteristics and all-cause mortality

| **Variable** | **HR (95% CI)^1^** | ***P*-value** |
| --- | --- | --- |
| Age (years) | 0.95 (0.94, 0.96) | 2.01x10^-20^ |
| Sex (% of males) | 1.79 (1.72, 1.87) | 2.47x10^-169^ |
| *Smoking status* |  |  |
| Never | reference |  |
| Former | 1.52 (1.45, 1.59) | 2.09x10^-72^ |
| Current | 3.26 (3.08, 3.45) | <1.20x10^-307^ |
| *Alcohol drinker status* |  |  |
| Never | reference |  |
| Former | 1.88 (1.66, 2.14) | 3.39x10^-22^ |
| Current | 0.93 (0.84, 1.03) | 0.17 |
| *Highest qualifications* |  |  |
| College or University degree | reference |  |
| A-levels | 1.10 (1.02, 1.19) | 0.02 |
| O-levels | 1.10 (1.03, 1.17) | 0.003 |
| CSEs | 1.34 (1.20, 1.51) | 3.38x10^-07^ |
| NVQ/HND/HNC | 1.35 (1.24, 1.46) | 3.27x10^-12^ |
| Other professional qualifications | 1.13 (1.03, 1.24) | 0.01 |
| *Current employment status* |  |  |
| In paid employment or self-employed | reference |  |
| Retired | 1.12 (1.06, 1.19) | 7.82x10^-05^ |
| Looking after home/family | 1.04 (0.87, 1.23) | 0.68 |
| Unable to work due to sickness/disability | 5.18 (4.80, 5.59) | <1.20x10^-307^ |
| Unemployed | 2.29 (1.93, 2.70) | 3.61x10^-22^ |
| Doing unpaid or voluntary work | 0.85 (0.58, 1.24) | 0.39 |
| Full or part-time student | 1.93 (1.12, 3.33) | 0.02 |
| Days/week spent doing vigorous physical activity | 0.94 (0.93, 0.95) | 1.40x10^-29^ |
| Genotyping chip^2^ | 1.34 (1.26, 1.42) | 2.21x10^-20^ |

*CI = confidence interval; CSE = certificate of secondary education; HNC = higher national certificate; HND = higher national diploma; HR = hazard ratio; NVQ = national vocational qualification*

*^1^Estimates represent the difference in hazards for all-cause mortality per unit increase in each continuous, categorical or binary variable in individuals of White British ancestry.*

*^2^There was evidence of differential array effect on markers scattered across the genome; therefore, the UK BiLEVE study genotyped on the Affymetrix Axiom Array was added as a covariable*

Table S6. Association of participants’ characteristics and the weighted GRS (comprising 77 SNPs)

| **Variable** | **N** | **Estimate (95% CI)^1^** | ***P*-value** |
| --- | --- | --- | --- |
| Age (years) | 335,308 | -0.002 (-0.004, 0.0003) | 0.09 |
| Sex (% of males) | 335,308 | 0.03 (-0.003, 0.07) | 0.07 |
| *Smoking status* | 334,142 |  |  |
| Never |  | reference |  |
| Former |  | 0.15 (0.11, 0.19) | 9.45x10^-13^ |
| Current |  | 0.23 (0.16, 0.29) | 6.68x10^-12^ |
| *Alcohol drinker status* | 335,074 |  |  |
| Never |  | reference |  |
| Former |  | 0.11 (-0.04, 0.25) | 0.16 |
| Current |  | -0.12 (-0.22, -0.01) | 0.04 |
| *Highest qualifications* | 275,544 |  |  |
| College or University degree |  | reference |  |
| A-levels |  | 0.01 (-0.05, 0.07) | 0.76 |
| O-levels |  | 0.10 (0.04, 0.15) | 2.88x10^-04^ |
| CSEs |  | 0.20 (0.11, 0.29) | 8.29x10^-06^ |
| NVQ/HND/HNC |  | 0.19 (0.11, 0.27) | 5.80x10^-06^ |
| Other professional qualifications |  | 0.10 (0.01, 0.19) | 0.02 |
| *Current employment status* | 332,835 |  |  |
| In paid employment or self-employed |  | reference |  |
| Retired |  | 0.02 (-0.03, 0.06) | 0.46 |
| Looking after home/family |  | -0.15 (-0.27, -0.03) | 0.01 |
| Unable to work due to sickness/disability |  | 0.30 (0.19, 0.41) | 1.28x10^-07^ |
| Unemployed |  | 0.03 (-0.14, 0.19) | 0.74 |
| Doing unpaid or voluntary work |  | 0.12 (-0.17, 0.41) | 0.42 |
| Full or part-time student |  | 0.07 (-0.37, 0.50) | 0.75 |
| Days/week spent doing vigorous physical activity | 319,813 | 0.01 (0.002, 0.02) | 0.02 |
| Genotyping chip^2^ | 335,308 | 0.15 (0.09, 0.22) | 3.47x10^-06^ |

*BMI = body mass index; CI = confidence interval; CSE = certificate of secondary education; GRS = genetic risk score; HNC = higher national certificate; HND = higher national diploma; NVQ = national vocational qualification; SNP = single nucleotide polymorphism*

*^1^Estimates represent the difference in the GRS (comprising 77 SNPs) per unit increase in each continuous, categorical or binary variable in individuals of White British ancestry adjusted for the first ten genetic principal components.*

*^2^There was evidence of differential array effect on markers scattered across the genome; therefore, the UK BiLEVE study genotyped on the Affymetrix Axiom Array was added as a covariable*

Table S7. MR analyses of higher BMI on mortality from all other causes in UK Biobank participants of White British ancestry

| **Sample** | **N^1^** | **Observational** | | | | | **MR-analyses** | | **DWH^5^** |
| --- | --- | --- | --- | --- | --- | --- | --- | --- | --- |
|  |  | **Unadjusted** | | | **Adjusted** | |  |  |  |
|  |  | **HR (95% CI)^2^** | ***P*-value** | **HR (95% CI)^3^** | | ***P*-value** | **HR (95% CI)^4^** | ***P*-value** |  |
| Whole sample | 1,099 | 1.02 (1.00, 1.03) | 0.01 | 1.01 (0.99, 1.02) | | 0.31 | 1.01 (0.90, 1.13) | 0.87 | 0.91 |
| Males | 696 | 1.00 (0.99, 1.02) | 0.65 | 0.99 (0.97, 1.01) | | 0.44 | 0.97 (0.84, 1.12) | 0.70 | 0.65 |
| Females | 403 | 1.02 (1.00, 1.04) | 0.04 | 1.02 (1.00, 1.04) | | 0.08 | 1.07 (0.89, 1.29) | 0.44 | 0.58 |

*BMI = body mass index; CI = confidence interval; HR = hazard ratio*

*^1^Number of deaths from all other causes*

*^2^Adjusted for secular trends (date of birth), estimates represent HR with each unit increase in BMI (kg/m2)*

*^3^Adjusted for secular trends (date of birth), highest household occupation, education, smoking status, alcohol intake and physical activity*

*^4^Adjusted for secular trends (date of birth) and the first ten genetic principal components*

*^5^P-value for comparing estimates derived from observational and MR analyses using a simplification of the matrix method for DWH test statistic (see Supporting Methods))*

Table S8a. Statistical test of the proportional hazards assumption in conventional Cox regression

| **Cause of death** | **Correlation coefficient (and corresponding *P*-values) for the test of proportional hazards^1^** | | | |
| --- | --- | --- | --- | --- |
|  | **Whole sample** | **Males** | **Females** |  |
| All-cause | -0.004 (0.76) | 0.003 (0.87) | -0.02 (0.42) |  |
| Cardiovascular disease | -0.08 (0.01) | -0.06 (0.08) | -0.12 (0.04) |  |
| Coronary heart disease | -0.01 (0.79) | 0.01 (0.75) | -0.10 (0.36) |  |
| Stroke | 0.03 (0.66) | 0.05 (0.57) | -0.005 (0.96) |  |
| Aortic aneurysm | -0.28 (0.02) | -0.30 (0.02) | - |  |
| Other cardiovascular diseases | -0.18 (0.003) | -0.17 (0.02) | -0.19 (0.08) |  |
| Respiratory diseases | -0.07 (0.22) | 0.01 (0.94) | -0.10 (0.36) |  |
| Cancer | 0.02 (0.23) | 0.03 (0.21) | 0.003 (0.89) |  |
| Lung cancer | 0.04 (0.36) | 0.15 (0.01) | -0.07 (0.25) |  |
| Prostate cancer | - | -0.06 (0.38) | - |  |
| Breast cancer | - | - | 0.10 (0.06) |  |
| Pre-menopausal | - | - | 0.23 (0.13) |  |
| Post-menopausal | - | - | 0.11 (0.05) |  |
| Colorectal cancer | -0.004 (0.93) | -0.001 (0.99) | -0.001 (0.99) |  |
| Pancreatic cancer | -0.10 (0.11) | -0.06 (0.45) | -0.13 (0.15) |  |
| Stomach cancer | 0.002 (0.98) | 0.12 (0.35) | - |  |
| Ovarian cancer | - | - | -0.10 (0.21) |  |
| Endometrial cancer | - | - | 0.27 (0.13) |  |
| Oesophageal cancer | -0.07 (0.33) | -0.08 (0.34) | 0.06 (0.70) |  |
| Malignant melanoma | -0.10 (0.34) | -0.05 (0.71) | -0.12 (0.51) |  |
| Kidney cancer | 0.09 (0.33) | 0.10 (0.35) | 0.09 (0.67) |  |
| Bladder cancer | -0.15 (0.24) | -0.12 (0.37) | - |  |
| Brain cancer | 0.04 (0.58) | 0.004 (0.97) | 0.11 (0.31) |  |
| Liver cancer | -0.02 (0.87) | -0.003 (0.98) | -0.09 (0.54) |  |
| Lymphatic cancer | -0.02 (0.69) | 0.005 (0.94) | -0.06 (0.46) |  |
| Other cancer | 0.08 (0.06) | 0.08 (0.15) | 0.09 (0.21) |  |
| External causes | 0.06 (0.33) | 0.04 (0.64) | 0.10 (0.37) |  |

*^1^Values represent the Pearson’s correlation coefficient (and corresponding P-values) between the first scaled Schoenfeld residual (obtained from the survival analysis of BMI on each mortality outcome adjusted for secular trends (date of birth), highest household occupation, education, smoking status, alcohol intake and physical activity) in conventional Cox regression and the rank-normalised natural logarithm of follow-up time (age). Positive/negative values suggest that the relationship between BMI and mortality increases/decreases over the follow-up period, respectively.*

Table S8b. Statistical test of the proportional hazards assumption using Mendelian randomization

| **Cause of death** | **Correlation coefficient (and corresponding *P*-values) for the test of proportional hazards^1^** | | |
| --- | --- | --- | --- |
|  | **Whole sample** | **Males** | **Females** |
| All-cause | 0.01 (0.22) | 0.02 (0.22) | 0.01 (0.57) |
| Cardiovascular disease | -0.003 (0.90) | 0.02 (0.50) | -0.05 (0.24) |
| Coronary heart disease | -0.02 (0.50) | -0.004 (0.91) | -0.09 (0.25) |
| Stroke | 0.08 (0.13) | 0.15 (0.04) | 0.001 (0.99) |
| Aortic aneurysm | 0.10 (0.29) | 0.15 (0.18) | - |
| Other cardiovascular diseases | -0.03 (0.56) | -0.01 (0.83) | -0.06 (0.51) |
| Respiratory diseases | -0.01 (0.90) | 0.03 (0.60) | -0.07 (0.39) |
| Cancer | 0.02 (0.09) | 0.02 (0.33) | 0.03 (0.16) |
| Lung cancer | -0.02 (0.49) | -0.01 (0.90) | -0.04 (0.39) |
| Prostate cancer | - | 0.12 (0.03) | - |
| Breast cancer | - | - | 0.01 (0.79) |
| Pre-menopausal | - | - | 0.07 (0.62) |
| Post-menopausal | - | - | -0.004 (0.93) |
| Colorectal cancer | 0.06 (0.13) | 0.02 (0.75) | 0.13 (0.05) |
| Pancreatic cancer | 0.06 (0.25) | 0.08 (0.24) | 0.04 (0.60) |
| Stomach cancer | 0.07 (0.39) | 0.05 (0.64) | - |
| Ovarian cancer | - | - | -0.11 (0.12) |
| Endometrial cancer | - | - | 0.14 (0.33) |
| Oesophageal cancer | -0.05 (0.41) | -0.04 (0.58) | -0.09 (0.52) |
| Malignant melanoma | 0.19 (0.04) | 0.24 (0.03) | 0.07 (0.64) |
| Kidney cancer | -0.04 (0.61) | -0.04 (0.68) | -0.07 (0.65) |
| Bladder cancer | 0.13 (0.18) | 0.12 (0.29) | - |
| Brain cancer | 0.01 (0.82) | -0.07 (0.35) | 0.16 (0.10) |
| Liver cancer | -0.14 (0.07) | -0.17 (0.08) | -0.09 (0.48) |
| Lymphatic cancer | -0.01 (0.85) | 0.01 (0.90) | -0.03 (0.70) |
| Other cancer | 0.05 (0.14) | 0.04 (0.36) | 0.08 (0.19) |
| External causes | 0.09 (0.12) | 0.08 (0.27) | 0.16 (0.12) |

*^1^Values represent the Pearson’s correlation coefficient (and corresponding P-values) between the first scaled Schoenfeld residual (obtained from the survival analysis of BMI on each mortality outcome adjusted for secular trends (date of birth) and the first ten genetic principal components) in Mendelian randomization analyses and the rank-normalised natural logarithm of follow-up time (age). Positive/negative values suggest that the relationship between BMI and mortality increases/decreases over the follow-up period, respectively.*

Table S9a. MR-Egger analysis of BMI on all-cause and cause-specific mortality in UK Biobank participants of White British ancestry

| **Cause of death** | **IVW** | | **Intercept of MR-Egger** | | **MR-Egger** | | **Weighted median** | | **Weighted mode** | |
| --- | --- | --- | --- | --- | --- | --- | --- | --- | --- | --- |
|  | **HR (95% CI)^1^** | ***P*** | **HR (95% CI)^1^** | ***P*** | **HR (95% CI)^1^** | ***P*** | **HR (95% CI)^1^** | ***P*** | **HR (95% CI)^1^** | ***P*** |
| All-cause | 1.02 (0.99, 1.06) | 0.25 | 1.00 (0.99, 1.01) | 0.72 | 1.04 (0.95, 1.13) | 0.43 | 1.04 (0.98, 1.08) | 0.26 | 1.04 (0.98, 1.11) | 0.16 |
| Cardiovascular disease | 1.07 (1.00, 1.15) | 0.05 | 1.00 (0.98, 1.02) | 0.90 | 1.06 (0.89, 1.26) | 0.49 | 1.08 (0.96, 1.18) | 0.24 | 1.20 (1.03, 1.39) | 0.02 |
| Coronary heart disease | 1.09 (0.99, 1.20) | 0.08 | 1.00 (0.97, 1.03) | 0.78 | 1.12 (0.89, 1.42) | 0.33 | 1.06 (0.94, 1.25) | 0.28 | 1.08 (0.90, 1.29) | 0.44 |
| Stroke | 0.98 (0.84, 1.15) | 0.83 | 1.02 (0.97, 1.08) | 0.41 | 0.85 (0.57, 1.26) | 0.40 | 0.92 (0.72, 1.14) | 0.41 | 0.82 (0.59, 1.14) | 0.24 |
| Aortic aneurysm | 0.86 (0.64, 1.16) | 0.33 | 1.05 (0.95, 1.15) | 0.34 | 0.62 (0.29, 1.31) | 0.21 | 0.80 (0.50, 1.24) | 0.30 | 0.82 (0.47, 1.44) | 0.49 |
| Other cardiovascular diseases | 1.18 (1.02, 1.35) | 0.02 | 0.99 (0.94, 1.03) | 0.52 | 1.30 (0.92, 1.84) | 0.13 | 1.18 (0.95, 1.52) | 0.13 | 1.22 (0.90, 1.66) | 0.20 |
| Respiratory diseases | 1.03 (0.90, 1.18) | 0.69 | 1.02 (0.98, 1.07) | 0.34 | 0.88 (0.63, 1.24) | 0.47 | 0.94 (0.79, 1.18) | 0.70 | 0.88 (0.67, 1.17) | 0.40 |
| Cancer | 0.99 (0.95, 1.03) | 0.69 | 1.00 (0.99, 1.01) | 0.98 | 0.99 (0.90, 1.10) | 0.90 | 1.01 (0.94, 1.07) | 0.86 | 1.01 (0.94, 1.10) | 0.76 |
| Lung cancer | 0.97 (0.89, 1.06) | 0.51 | 1.02 (0.99, 1.05) | 0.24 | 0.86 (0.68, 1.08) | 0.18 | 0.95 (0.83, 1.11) | 0.51 | 0.94 (0.77, 1.14) | 0.51 |
| Colorectal cancer | 1.05 (0.91, 1.20) | 0.49 | 1.02 (0.97, 1.06) | 0.48 | 0.94 (0.67, 1.32) | 0.71 | 1.07 (0.87, 1.29) | 0.56 | 1.09 (0.86, 1.39) | 0.47 |
| Pancreatic cancer | 1.07 (0.92, 1.25) | 0.35 | 1.03 (0.98, 1.08) | 0.25 | 0.88 (0.60, 1.28) | 0.51 | 1.00 (0.82, 1.31) | 0.81 | 1.01 (0.77, 1.34) | 0.93 |
| Stomach cancer | 1.15 (0.91, 1.46) | 0.24 | 0.94 (0.87, 1.02) | 0.13 | 1.74 (0.97, 3.11) | 0.06 | 1.58 (0.94, 1.97) | 0.10 | 1.68 (0.99, 2.86) | 0.06 |
| Oesophageal cancer | 1.17 (0.98, 1.39) | 0.08 | 0.99 (0.93, 1.04) | 0.66 | 1.28 (0.83, 1.97) | 0.26 | 1.00 (0.83, 1.47) | 0.49 | 1.02 (0.70, 1.49) | 0.91 |
| Malignant melanoma | 1.15 (0.88, 1.51) | 0.31 | 1.04 (0.95, 1.13) | 0.38 | 0.87 (0.44, 1.71) | 0.69 | 0.89 (0.68, 1.56) | 0.89 | 1.02 (0.62, 1.69) | 0.94 |
| Kidney cancer | 0.95 (0.77, 1.18) | 0.65 | 1.03 (0.96, 1.10) | 0.46 | 0.79 (0.47, 1.35) | 0.39 | 1.02 (0.67, 1.39) | 0.85 | 0.92 (0.56, 1.50) | 0.73 |
| Bladder cancer | 0.84 (0.63, 1.12) | 0.23 | 0.95 (0.87, 1.05) | 0.31 | 1.17 (0.58, 2.37) | 0.65 | 0.94 (0.57, 1.43) | 0.73 | 1.09 (0.61, 1.96) | 0.76 |
| Brain cancer | 1.02 (0.85, 1.23) | 0.80 | 1.01 (0.95, 1.07) | 0.76 | 0.96 (0.61, 1.52) | 0.86 | 1.12 (0.80, 1.48) | 0.57 | 1.18 (0.78, 1.78) | 0.45 |
| Liver cancer | 1.00 (0.80, 1.25) | 0.98 | 1.03 (0.96, 1.10) | 0.46 | 0.83 (0.47, 1.44) | 0.50 | 0.94 (0.66, 1.36) | 0.73 | 1.08 (0.69, 1.71) | 0.73 |
| Lymphatic cancer | 1.03 (0.91, 1.17) | 0.62 | 1.00 (0.96, 1.04) | 0.98 | 1.03 (0.76, 1.40) | 0.86 | 1.04 (0.86, 1.28) | 0.64 | 0.99 (0.76, 1.29) | 0.94 |
| Other cancers | 0.96 (0.87, 1.07) | 0.47 | 0.96 (0.93, 1.00) | 0.03 | 1.25 (0.97, 1.62) | 0.09 | 1.00 (0.89, 1.20) | 0.68 | 1.04 (0.85, 1.27) | 0.69 |
| External causes | 1.23 (1.03, 1.46) | 0.03 | 1.04 (0.98, 1.10) | 0.22 | 0.95 (0.61, 1.48) | 0.83 | 1.07 (0.84, 1.45) | 0.49 | 1.05 (0.75, 1.48) | 0.76 |

*BMI = body mass index; CI = confidence interval; HR = hazard ratio; IVW = inverse variance weighted; MR = Mendelian randomization*

*^1^Adjusted for secular trends (date of birth) and the first ten genetic principal components, estimates represent HR with each unit increase in BMI (kg/m^2^)*

Table S9b. MR-Egger analysis of BMI on all-cause and cause-specific mortality in UK Biobank males of White British ancestry

| **Cause of death** | **IVW** | | **Intercept of MR-Egger** | | **MR-Egger** | | **Weighted median** | | **Weighted mode** | |
| --- | --- | --- | --- | --- | --- | --- | --- | --- | --- | --- |
|  | **HR (95% CI)^1^** | ***P*** | **HR (95% CI)^1^** | ***P*** | **HR (95% CI)^1^** | ***P*** | **HR (95% CI)^1^** | ***P*** | **HR (95% CI)^1^** | ***P*** |
| All-cause | 1.02 (0.98, 1.06) | 0.28 | 0.99 (0.98, 1.00) | 0.13 | 1.09 (0.99, 1.20) | 0.07 | 1.02 (0.97, 1.11) | 0.29 | 1.03 (0.95, 1.12) | 0.42 |
| Cardiovascular disease | 1.07 (0.99, 1.15) | 0.11 | 0.99 (0.97, 1.02) | 0.54 | 1.12 (0.93, 1.36) | 0.23 | 1.04 (0.93, 1.19) | 0.43 | 1.04 (0.88, 1.23) | 0.65 |
| Coronary heart disease | 1.09 (0.98, 1.21) | 0.11 | 0.99 (0.96, 1.02) | 0.51 | 1.18 (0.91, 1.53) | 0.21 | 1.07 (0.94, 1.28) | 0.32 | 1.10 (0.90, 1.35) | 0.35 |
| Stroke | 1.00 (0.81, 1.23) | 0.98 | 1.00 (0.94, 1.07) | 0.92 | 0.98 (0.58, 1.65) | 0.93 | 0.98 (0.70, 1.26) | 0.71 | 0.91 (0.59, 1.40) | 0.66 |
| Aortic aneurysm | 0.86 (0.62, 1.21) | 0.39 | 1.06 (0.95, 1.18) | 0.29 | 0.57 (0.25, 1.33) | 0.19 | 0.66 (0.48, 1.33) | 0.37 | 0.71 (0.38, 1.32) | 0.28 |
| Other cardiovascular diseases | 1.11 (0.93, 1.33) | 0.24 | 0.97 (0.92, 1.03) | 0.35 | 1.35 (0.86, 2.11) | 0.19 | 1.14 (0.86, 1.52) | 0.29 | 1.14 (0.81, 1.62) | 0.45 |
| Respiratory diseases | 1.03 (0.88, 1.20) | 0.69 | 1.02 (0.97, 1.07) | 0.52 | 0.92 (0.63, 1.35) | 0.67 | 1.07 (0.77, 1.23) | 0.83 | 0.87 (0.62, 1.22) | 0.42 |
| Cancer | 1.00 (0.95, 1.06) | 0.98 | 0.99 (0.97, 1.01) | 0.30 | 1.07 (0.93, 1.23) | 0.33 | 1.03 (0.94, 1.12) | 0.58 | 1.02 (0.91, 1.15) | 0.69 |
| Lung cancer | 0.94 (0.83, 1.06) | 0.32 | 1.01 (0.97, 1.05) | 0.52 | 0.86 (0.63, 1.17) | 0.32 | 0.89 (0.77, 1.13) | 0.47 | 0.85 (0.65, 1.11) | 0.24 |
| Prostate cancer | 0.91 (0.76, 1.09) | 0.32 | 0.96 (0.91, 1.02) | 0.20 | 1.19 (0.76, 1.86) | 0.43 | 1.01 (0.76, 1.25) | 0.81 | 1.05 (0.76, 1.45) | 0.77 |
| Colorectal cancer | 1.07 (0.91, 1.26) | 0.40 | 1.01 (0.96, 1.06) | 0.69 | 0.99 (0.66, 1.49) | 0.98 | 1.00 (0.81, 1.34) | 0.82 | 1.04 (0.73, 1.49) | 0.82 |
| Pancreatic cancer | 1.14 (0.93, 1.39) | 0.21 | 1.02 (0.96, 1.09) | 0.45 | 0.96 (0.58, 1.58) | 0.86 | 0.96 (0.76, 1.44) | 0.80 | 0.98 (0.66, 1.45) | 0.93 |
| Stomach cancer | 1.12 (0.84, 1.50) | 0.42 | 0.98 (0.89, 1.07) | 0.63 | 1.32 (0.64, 2.70) | 0.45 | 1.45 (0.77, 2.04) | 0.33 | 1.62 (0.86, 3.06) | 0.14 |
| Oesophageal cancer | 1.21 (0.99, 1.48) | 0.06 | 0.97 (0.91, 1.04) | 0.43 | 1.45 (0.89, 2.38) | 0.14 | 1.17 (0.86, 1.62) | 0.31 | 1.33 (0.85, 2.09) | 0.22 |
| Malignant melanoma^2^ | 1.03 (0.74, 1.43) | 0.85 | 1.02 (0.92, 1.13) | 0.73 | 0.91 (0.40, 2.05) | 0.81 | 1.14 (0.64, 1.76) | 0.76 | 1.14 (0.61, 2.11) | 0.68 |
| Kidney cancer | 1.04 (0.79, 1.36) | 0.77 | 1.02 (0.94, 1.11) | 0.66 | 0.91 (0.46, 1.78) | 0.77 | 1.06 (0.65, 1.47) | 0.98 | 0.92 (0.55, 1.57) | 0.77 |
| Bladder cancer | 0.80 (0.58, 1.10) | 0.17 | 0.93 (0.84, 1.03) | 0.15 | 1.36 (0.61, 3.06) | 0.45 | 0.90 (0.53, 1.53) | 0.68 | 1.04 (0.54, 1.99) | 0.91 |
| Brain cancer | 1.12 (0.90, 1.40) | 0.29 | 1.01 (0.95, 1.09) | 0.69 | 1.02 (0.59, 1.75) | 0.96 | 1.16 (0.74, 1.66) | 0.58 | 1.33 (0.79, 2.21) | 0.28 |
| Liver cancer | 1.04 (0.78, 1.39) | 0.78 | 0.98 (0.89, 1.07) | 0.65 | 1.21 (0.59, 2.47) | 0.60 | 1.29 (0.76, 1.86) | 0.46 | 1.35 (0.75, 2.44) | 0.32 |
| Lymphatic cancer | 1.02 (0.87, 1.19) | 0.80 | 0.99 (0.94, 1.04) | 0.61 | 1.12 (0.76, 1.65) | 0.57 | 1.02 (0.86, 1.39) | 0.45 | 1.18 (0.85, 1.63) | 0.32 |
| Other cancers | 0.92 (0.80, 1.05) | 0.22 | 0.95 (0.91, 0.99) | 0.01 | 1.35 (0.97, 1.89) | 0.07 | 1.06 (0.83, 1.28) | 0.81 | 1.08 (0.84, 1.38) | 0.57 |
| External causes | 1.09 (0.88, 1.34) | 0.42 | 1.05 (0.98, 1.12) | 0.16 | 0.78 (0.47, 1.30) | 0.34 | 0.90 (0.71, 1.32) | 0.84 | 0.92 (0.62, 1.36) | 0.67 |

*BMI = body mass index; CI = confidence interval; HR = hazard ratio; IVW = inverse variance weighted; MR = Mendelian randomization*

*^1^Adjusted for secular trends (date of birth) and the first ten genetic principal components, estimates represent HR with each unit increase in BMI (kg/m^2^)*

*^2^Estimates obtained for the causal effect of BMI on mortality from malignant melanoma in males only used 76/77 SNPs, as all men who died from malignant melanoma had a dosage of ‘0’ for one SNP (rs17024393); thus, providing no variation in mortality risk with this SNP.*

Table S9c. MR-Egger analysis of BMI on all-cause and cause-specific mortality in UK Biobank females of White British ancestry

| **Cause of death** | **IVW** | | **Intercept of MR-Egger** | | **MR-Egger** | | **Weighted median** | | **Weighted mode** | |
| --- | --- | --- | --- | --- | --- | --- | --- | --- | --- | --- |
|  | **HR (95% CI)^1^** | ***P*** | **HR (95% CI)^1^** | ***P*** | **HR (95% CI)^1^** | ***P*** | **HR (95% CI)^1^** | ***P*** | **HR (95% CI)^1^** | ***P*** |
| All-cause | 1.02 (0.96, 1.08) | 0.50 | 1.01 (0.99, 1.03) | 0.27 | 0.95 (0.82, 1.09) | 0.45 | 1.00 (0.93, 1.09) | 0.83 | 1.01 (0.92, 1.12) | 0.80 |
| Cardiovascular disease | 1.09 (0.94, 1.26) | 0.27 | 1.03 (0.98, 1.08) | 0.23 | 0.88 (0.61, 1.28) | 0.51 | 0.90 (0.80, 1.22) | 0.89 | 0.85 (0.64, 1.12) | 0.25 |
| Coronary heart disease | 1.10 (0.88, 1.37) | 0.40 | 1.03 (0.96, 1.11) | 0.34 | 0.86 (0.50, 1.49) | 0.58 | 1.05 (0.74, 1.45) | 0.82 | 1.25 (0.81, 1.93) | 0.31 |
| Stroke | 0.95 (0.72, 1.24) | 0.68 | 1.05 (0.96, 1.14) | 0.31 | 0.69 (0.35, 1.35) | 0.27 | 0.85 (0.61, 1.27) | 0.48 | 0.87 (0.54, 1.41) | 0.57 |
| Other cardiovascular diseases | 1.31 (1.01, 1.71) | 0.05 | 1.01 (0.93, 1.10) | 0.85 | 1.24 (0.64, 2.40) | 0.53 | 1.29 (0.86, 1.96) | 0.27 | 1.43 (0.84, 2.44) | 0.19 |
| Respiratory diseases | 1.03 (0.83, 1.28) | 0.80 | 1.03 (0.96, 1.11) | 0.39 | 0.83 (0.48, 1.43) | 0.49 | 0.87 (0.65, 1.35) | 0.68 | 0.89 (0.56, 1.39) | 0.60 |
| Cancer | 0.98 (0.92, 1.04) | 0.54 | 1.01 (0.99, 1.03) | 0.25 | 0.91 (0.78, 1.05) | 0.19 | 0.96 (0.89, 1.08) | 0.63 | 0.97 (0.87, 1.09) | 0.61 |
| Lung cancer | 1.02 (0.88, 1.17) | 0.83 | 1.02 (0.98, 1.07) | 0.34 | 0.87 (0.62, 1.23) | 0.43 | 0.95 (0.78, 1.19) | 0.70 | 0.90 (0.68, 1.21) | 0.51 |
| Breast cancer | 0.87 (0.76, 0.99) | 0.03 | 0.98 (0.94, 1.03) | 0.41 | 0.98 (0.71, 1.37) | 0.92 | 0.92 (0.73, 1.10) | 0.34 | 0.92 (0.70, 1.21) | 0.54 |
| Pre-menopausal | 0.83 (0.55, 1.27) | 0.39 | 0.99 (0.86, 1.13) | 0.85 | 0.92 (0.32, 2.61) | 0.87 | 0.94 (0.45, 1.65) | 0.69 | 1.01 (0.46, 2.22) | 0.98 |
| Post-menopausal | 0.87 (0.76, 1.00) | 0.05 | 0.98 (0.94, 1.03) | 0.42 | 0.99 (0.70, 1.41) | 0.97 | 0.91 (0.72, 1.13) | 0.35 | 0.92 (0.68, 1.23) | 0.57 |
| Colorectal cancer | 1.01 (0.80, 1.28) | 0.92 | 1.03 (0.95, 1.11) | 0.51 | 0.85 (0.47, 1.53) | 0.58 | 1.26 (0.81, 1.52) | 0.44 | 1.30 (0.82, 2.05) | 0.26 |
| Pancreatic cancer | 1.02 (0.82, 1.27) | 0.87 | 1.03 (0.96, 1.11) | 0.38 | 0.81 (0.47, 1.41) | 0.46 | 1.08 (0.73, 1.47) | 0.79 | 1.12 (0.76, 1.65) | 0.58 |
| Ovarian cancer | 1.16 (0.94, 1.42) | 0.16 | 1.03 (0.96, 1.10) | 0.39 | 0.95 (0.57, 1.57) | 0.83 | 1.17 (0.83, 1.55) | 0.45 | 1.20 (0.82, 1.76) | 0.35 |
| Endometrial cancer | 0.73 (0.49, 1.11) | 0.14 | 1.02 (0.90, 1.17) | 0.73 | 0.62 (0.22, 1.76) | 0.36 | 0.58 (0.33, 1.12) | 0.12 | 0.43 (0.17, 1.05) | 0.07 |
| Oesophageal cancer | 1.03 (0.70, 1.51) | 0.88 | 1.03 (0.91, 1.17) | 0.60 | 0.82 (0.31, 2.13) | 0.67 | 0.89 (0.48, 1.59) | 0.70 | 0.83 (0.35, 1.97) | 0.68 |
| Malignant melanoma | 1.44 (0.91, 2.26) | 0.11 | 1.07 (0.92, 1.24) | 0.37 | 0.89 (0.29, 2.78) | 0.85 | 1.41 (0.58, 2.14) | 0.75 | 1.06 (0.41, 2.71) | 0.91 |
| Kidney cancer | 0.77 (0.50, 1.18) | 0.22 | 1.04 (0.90, 1.19) | 0.58 | 0.58 (0.20, 1.70) | 0.32 | 0.84 (0.38, 1.58) | 0.57 | 0.80 (0.29, 2.21) | 0.67 |
| Brain cancer | 0.90 (0.66, 1.22) | 0.50 | 1.00 (0.90, 1.10) | 0.93 | 0.93 (0.43, 2.00) | 0.85 | 1.03 (0.62, 1.69) | 0.88 | 1.05 (0.58, 1.90) | 0.87 |
| Liver cancer | 0.96 (0.67, 1.39) | 0.84 | 1.10 (0.98, 1.24) | 0.10 | 0.47 (0.19, 1.18) | 0.11 | 0.67 (0.41, 1.25) | 0.27 | 0.47 (0.22, 1.01) | 0.06 |
| Lymphatic cancer | 1.05 (0.86, 1.29) | 0.61 | 1.02 (0.96, 1.09) | 0.53 | 0.91 (0.55, 1.51) | 0.70 | 0.91 (0.70, 1.30) | 0.79 | 0.86 (0.55, 1.33) | 0.49 |
| Other cancers | 1.03 (0.88, 1.22) | 0.69 | 0.99 (0.94, 1.04) | 0.70 | 1.11 (0.74, 1.68) | 0.61 | 1.07 (0.85, 1.41) | 0.44 | 1.10 (0.77, 1.57) | 0.60 |
| External causes | 1.59 (1.19, 2.12) | 0.002 | 1.01 (0.92, 1.11) | 0.77 | 1.44 (0.71, 2.94) | 0.31 | 1.40 (0.89, 2.26) | 0.10 | 1.37 (0.78, 2.42) | 0.28 |

*BMI = body mass index; CI = confidence interval; HR = hazard ratio; IVW = inverse variance weighted; MR = Mendelian randomization*

*^1^Adjusted for secular trends (date of birth) and the first ten genetic principal components, estimates represent HR with each unit increase in BMI (kg/m^2^)*

Table S10a. Association between the weighted GRS (comprising 77 SNPs) and BMI in UK Biobank participants of White British ancestry additionally adjusting for confounding factors

| **Sample** | **N** | **Effect estimate**  **(95% CI)^1^** | ***P*-value** | **R^2^ (%)^2^** |
| --- | --- | --- | --- | --- |
| Whole sample | 265,633 | 0.107 (0.104, 0.110) | <1.20x10^-307^ | 5.40 |
| Males | 123,509 | 0.099 (0.095, 0.103) | <1.20x10^-307^ | 6.22 |
| Females | 142,124 | 0.114 (0.109, 0.118) | <1.20x10^-307^ | 5.52 |

*BMI = body mass index; CI = confidence interval; GRS = genetic risk score; SNP = single nucleotide polymorphism*

*^1^Effect estimate (and corresponding P-value) represents the change in BMI (kg/m^2^) per BMI-increasing allele in individuals of White British ancestry adjusted for the first ten genetic principal components, highest household occupation, education, smoking status, alcohol intake, physical activity and genotyping chip*

*^2^Variance in BMI explained by the GRS*

Table S10b. MR analyses of all-cause and cause-specific mortality by BMI in UK Biobank participants of White British ancestry additionally adjusting for confounding factors

| **Cause of death** | **Whole sample** | | **Males** | | **Females** | |
| --- | --- | --- | --- | --- | --- | --- |
|  | **HR (95% CI)^1^** | ***P*-value** | **HR (95% CI)^1^** | ***P*-value** | **HR (95% CI)^1^** | ***P*-value** |
| All-cause | 1.02 (0.97, 1.07) | 0.45 | 1.03 (0.96, 1.10) | 0.41 | 1.00 (0.93, 1.08) | 0.91 |
| Cardiovascular disease | 1.14 (1.02, 1.27) | 0.02 | 1.12 (0.98, 1.27) | 0.10 | 1.22 (0.97, 1.53) | 0.09 |
| Coronary heart disease | 1.22 (1.05, 1.42) | 0.01 | 1.23 (1.04, 1.45) | 0.02 | 1.24 (0.83, 1.85) | 0.30 |
| Stroke | 0.98 (0.75, 1.29) | 0.89 | 0.88 (0.61, 1.29) | 0.52 | 1.12 (0.75, 1.68) | 0.59 |
| Aortic aneurysm | 0.85 (0.54, 1.32) | 0.47 | 0.80 (0.48, 1.35) | 0.41 | - | - |
| Other cardiovascular diseases | 1.15 (0.92, 1.45) | 0.23 | 1.07 (0.80, 1.42) | 0.66 | 1.37 (0.91, 2.06) | 0.13 |
| Respiratory diseases | 0.86 (0.68, 1.09) | 0.21 | 0.82 (0.61, 1.10) | 0.18 | 0.95 (0.63, 1.42) | 0.79 |
| Cancer | 0.97 (0.91, 1.04) | 0.41 | 1.00 (0.91, 1.09) | 0.94 | 0.95 (0.87, 1.04) | 0.25 |
| Lung cancer | 0.96 (0.81, 1.13) | 0.60 | 0.95 (0.76, 1.19) | 0.68 | 0.96 (0.75, 1.23) | 0.76 |
| Prostate cancer | - | - | 0.70 (0.53, 0.93) | 0.01 | - | - |
| Breast cancer | - | - | - | - | 0.84 (0.69, 1.03) | 0.09 |
| Pre-menopausal | - | - | - | - | 0.77 (0.44, 1.37) | 0.38 |
| Post-menopausal | - | - | - | - | 0.85 (0.69, 1.05) | 0.14 |
| Colorectal cancer | 1.12 (0.92, 1.35) | 0.25 | 1.19 (0.92, 1.54) | 0.18 | 1.03 (0.78, 1.37) | 0.84 |
| Pancreatic cancer | 1.02 (0.81, 1.30) | 0.84 | 1.04 (0.74, 1.46) | 0.81 | 1.01 (0.72, 1.40) | 0.97 |
| Stomach cancer | 1.36 (0.89, 2.07) | 0.15 | 1.36 (0.82, 2.26) | 0.23 | - | - |
| Ovarian cancer | - | - | - | - | 1.16 (0.86, 1.57) | 0.33 |
| Endometrial cancer | - | - | - | - | 0.67 (0.35, 1.28) | 0.23 |
| Oesophageal cancer | 1.09 (0.82, 1.45) | 0.55 | 1.22 (0.87, 1.71) | 0.25 | 0.77 (0.43, 1.38) | 0.39 |
| Malignant melanoma | 1.08 (0.72, 1.60) | 0.72 | 0.91 (0.54, 1.52) | 0.71 | 1.41 (0.74, 2.68) | 0.29 |
| Kidney cancer | 0.96 (0.66, 1.38) | 0.81 | 1.09 (0.70, 1.68) | 0.70 | 0.67 (0.32, 1.37) | 0.27 |
| Bladder cancer | 0.82 (0.51, 1.31) | 0.41 | 0.89 (0.52, 1.52) | 0.66 | - | - |
| Brain cancer | 1.01 (0.77, 1.32) | 0.95 | 1.34 (0.93, 1.92) | 0.12 | 0.71 (0.47, 1.05) | 0.09 |
| Liver cancer | 0.94 (0.65, 1.36) | 0.75 | 0.79 (0.48, 1.29) | 0.34 | 1.21 (0.69, 2.11) | 0.51 |
| Lymphatic cancer | 1.03 (0.84, 1.26) | 0.77 | 1.05 (0.80, 1.36) | 0.74 | 1.01 (0.73, 1.40) | 0.94 |
| Other cancers | 0.95 (0.80, 1.12) | 0.52 | 0.89 (0.71, 1.12) | 0.32 | 1.02 (0.78, 1.34) | 0.87 |
| External causes | 1.41 (1.10, 1.81) | 0.01 | 1.28 (0.93, 1.77) | 0.13 | 1.71 (1.12, 2.59) | 0.01 |

*BMI = body mass index; CI = confidence interval; HR = hazard ratio*

*^1^Adjusted for secular trends (date of birth), the first ten genetic principal components, highest household occupation, education, smoking status, alcohol intake, physical activity and genotyping chip; estimates represent HR with each unit increase in BMI (kg/m^2^)*

Table S11a. Association between the weighted GRS (comprising 70 SNPs, after excluding 7 genetic variants implicated as pleiotropic) and BMI in UK Biobank participants of White British ancestry

| **Sample** | **N** | **Effect estimate**  **(95% CI)^1^** | ***P*-value** | **R^2^ (%)^2^** |
| --- | --- | --- | --- | --- |
| Whole sample | 335,308 | 0.112 (0.109, 0.115) | <1.20x10^-307^ | 1.68 |
| Males | 154,967 | 0.105 (0.101, 0.109) | <1.20x10^-307^ | 1.90 |
| Females | 180,341 | 0.117 (0.112, 0.121) | <1.20x10^-307^ | 1.57 |

*BMI = body mass index; CI = confidence interval; GRS = genetic risk score; SNP = single nucleotide polymorphism*

*^1^Effect estimate (and corresponding P-value) represents the change in BMI (kg/m^2^) per BMI-increasing allele in individuals of White British ancestry adjusted for the first ten genetic principal components*

*^2^Variance in BMI explained by the GRS*

Table S11b. MR analyses of all-cause and cause-specific mortality by BMI in UK Biobank participants of White British ancestry using a GRS (comprising 70 SNPs, after excluding 7 genetic variants implicated as pleiotropic)

| **Cause of death** | **Whole sample** | | **Males** | | **Females** | |
| --- | --- | --- | --- | --- | --- | --- |
|  | **HR (95% CI)^1^** | ***P*-value** | **HR (95% CI)^1^** | ***P*-value** | **HR (95% CI)^1^** | ***P*-value** |
| All-cause | 1.03 (0.99, 1.07) | 0.21 | 1.03 (0.98, 1.08) | 0.29 | 1.02 (0.96, 1.09) | 0.48 |
| Cardiovascular disease | 1.11 (1.01, 1.21) | 0.02 | 1.10 (0.99, 1.22) | 0.09 | 1.14 (0.96, 1.36) | 0.13 |
| Coronary heart disease | 1.14 (1.02, 1.29) | 0.03 | 1.13 (0.99, 1.30) | 0.06 | 1.20 (0.90, 1.60) | 0.22 |
| Stroke | 0.97 (0.78, 1.19) | 0.75 | 1.03 (0.77, 1.37) | 0.86 | 0.90 (0.66, 1.23) | 0.51 |
| Aortic aneurysm | 0.78 (0.54, 1.14) | 0.20 | 0.81 (0.52, 1.26) | 0.36 | - | - |
| Other cardiovascular disease | 1.24 (1.03, 1.50) | 0.03 | 1.12 (0.88, 1.42) | 0.36 | 1.53 (1.10, 2.11) | 0.01 |
| Respiratory diseases | 1.06 (0.90, 1.26) | 0.47 | 1.06 (0.86, 1.31) | 0.57 | 1.07 (0.80, 1.44) | 0.66 |
| Cancer | 0.98 (0.93, 1.03) | 0.44 | 0.99 (0.92, 1.07) | 0.84 | 0.96 (0.89, 1.04) | 0.35 |
| Lung cancer | 0.95 (0.84, 1.08) | 0.46 | 0.91 (0.77, 1.07) | 0.25 | 1.02 (0.85, 1.23) | 0.84 |
| Prostate cancer | - | - | 0.84 (0.67, 1.05) | 0.13 | - | - |
| Breast cancer | - | - | - | - | 0.81 (0.68, 0.97) | 0.02 |
| Pre-menopausal | - | - | - | - | 0.87 (0.50, 1.53) | 0.64 |
| Post-menopausal | - | - | - | - | 0.80 (0.67, 0.97) | 0.02 |
| Colorectal cancer | 1.05 (0.89, 1.24) | 0.59 | 1.06 (0.85, 1.33) | 0.59 | 1.02 (0.79, 1.32) | 0.87 |
| Pancreatic cancer | 1.04 (0.85, 1.27) | 0.70 | 1.12 (0.85, 1.49) | 0.42 | 0.96 (0.72, 1.28) | 0.78 |
| Stomach cancer | 1.22 (0.88, 1.69) | 0.23 | 1.19 (0.80, 1.75) | 0.39 | - | - |
| Ovarian cancer | - | - | - | - | 1.16 (0.89, 1.51) | 0.28 |
| Endometrial cancer | - | - | - | - | 0.60 (0.35, 1.04) | 0.07 |
| Oesophageal cancer | 1.23 (0.98, 1.56) | 0.08 | 1.29 (0.99, 1.68) | 0.06 | 1.07 (0.64, 1.79) | 0.79 |
| Malignant melanoma | 1.24 (0.86, 1.77) | 0.24 | 1.03 (0.65, 1.62) | 0.90 | 1.74 (0.95, 3.18) | 0.07 |
| Kidney cancer | 0.93 (0.69, 1.24) | 0.62 | 1.03 (0.73, 1.44) | 0.88 | 0.68 (0.38, 1.23) | 0.20 |
| Bladder cancer | 0.79 (0.53, 1.17) | 0.23 | 0.75 (0.47, 1.18) | 0.21 | - | - |
| Brain cancer | 1.03 (0.81, 1.30) | 0.83 | 1.16 (0.85, 1.57) | 0.36 | 0.86 (0.60, 1.25) | 0.44 |
| Liver cancer | 0.98 (0.72, 1.32) | 0.88 | 1.06 (0.71, 1.59) | 0.76 | 0.87 (0.55, 1.39) | 0.56 |
| Lymphatic cancer | 1.04 (0.88, 1.24) | 0.63 | 1.09 (0.87, 1.35) | 0.46 | 0.98 (0.74, 1.29) | 0.87 |
| Other cancer | 0.93 (0.81, 1.07) | 0.31 | 0.85 (0.71, 1.03) | 0.10 | 1.05 (0.84, 1.31) | 0.67 |
| External causes | 1.30 (1.04, 1.63) | 0.02 | 1.13 (0.86, 1.49) | 0.39 | 1.74 (1.18, 2.56) | 0.005 |

*BMI = body mass index; CI = confidence interval; HR = hazard ratio*

*^1^Adjusted for secular trends (date of birth) and the first ten genetic principal components; estimates represent HR with each unit increase in BMI (kg/m^2^)*

Figure S1. Exclusions made based on in-house QC parameters

*
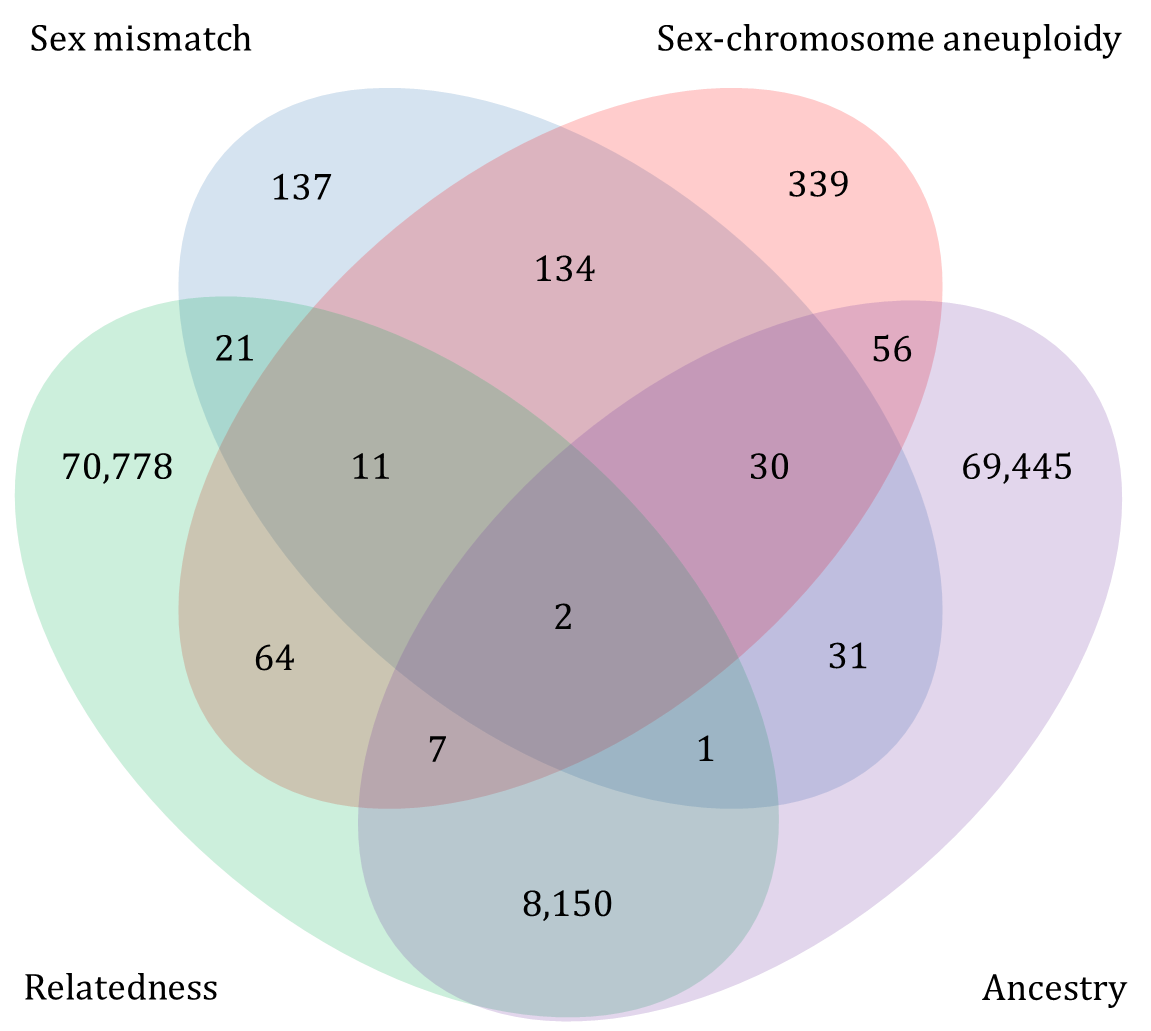
*

Figure S2. MR-Egger analysis for mortality outcomes with any evidence for pleiotropy


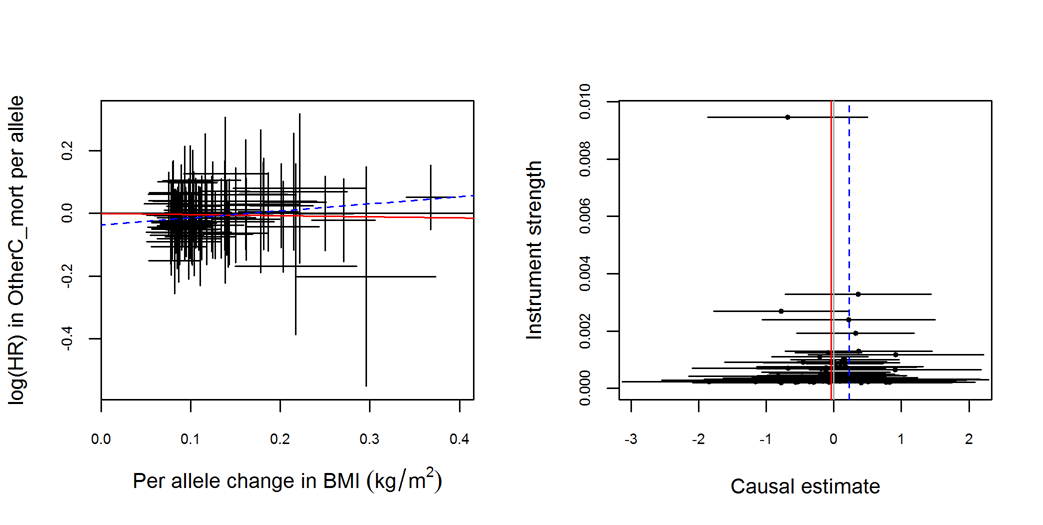


(a) other cancers in the whole UK Biobank sample


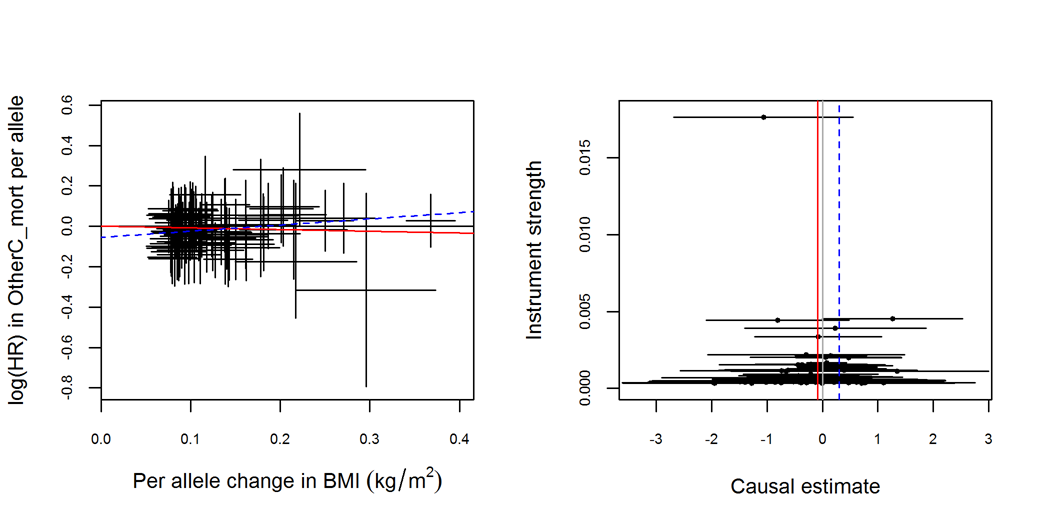


(b) other cancers in males only

***Supporting References***

1. Collins R. What makes UK Biobank special? *The Lancet*. 2012;379:1173-1174.

2. Bycroft C, Freeman C, Petkova D, *et al*. Genome-wide genetic data on ~500,000 UK Biobank participants. *bioRxiv*. 2017; doi:10.1101/166298.

3. Allen NE, Sudlow C, Peakman T and Collins R. UK Biobank Data: Come and Get It. *Science Translational Medicine*. 2014;6:224ed4.

4. Tyrrell J, Jones SE, Beaumont R, *et al*. Height, body mass index, and socioeconomic status: mendelian randomisation study in UK Biobank. *BMJ*. 2016;352.

5. Mitchell R, Hemani G, Dudding T, Paternoster L. UK Biobank Genetic Data: MRC-IEU Quality Control, Version 1. 2017.

6. Locke AE, Kahali B, Berndt SI, et al. Genetic studies of body mass index yield new insights for obesity biology. Nature. 2015;518:197-206.

7. Greene WH. *Econometric Analysis, 7th Edition*: Pearson; 2012.

8. Bowden J, Davey Smith G and Burgess S. Mendelian randomization with invalid instruments: effect estimation and bias detection through Egger regression. *International Journal of Epidemiology*. 2015;44(2):512-525.

9. Bowden J, Davey Smith G, Haycock PC and Burgess S. Consistent Estimation in Mendelian Randomization with Some Invalid Instruments Using a Weighted Median Estimator. *Genetic Epidemiology*. 2016;40:304-314.

10. Hartwig FP, Davey Smith G and Bowden J. Robust inference in summary data Mendelian randomization via the zero modal pleiotropy assumption. *International Journal of Epidemiology*. 2017;46:1985-1998

11. Bowden J, Burgess S and Davey Smith G. Response to Hartwig and Davies. International Journal of Epidemiology. 2016;45:1679-1680.

12. Hartwig FP and Davies NM. Why internal weights should be avoided (not only) in MR-Egger regression. International Journal of Epidemiology. 2016;45:1676-1678.

13. Corbin LJ, Richmond RC, Wade KH, *et al*. BMI as a Modifiable Risk Factor for Type 2 Diabetes: Refining and Understanding Causal Estimates Using Mendelian Randomization. *Diabetes*. 2016;65:3002-3007.

14. Yaghootkar H, Lotta LA, Tyrrell J, *et al*. Genetic Evidence for a Link Between Favorable Adiposity and Lower Risk of Type 2 Diabetes, Hypertension, and Heart Disease. *Diabetes*. 2016;65:2448.
